# A novel and selective fluorescent ligand for the study of adenosine A_2B_ receptors

**DOI:** 10.1002/prp2.1223

**Published:** 2024-06-21

**Authors:** Foteini Patera, Sarah J. Mistry, Nicholas D. Kindon, Eleonora Comeo, Joelle Goulding, Barrie Kellam, Laura E. Kilpatrick, Hester Franks, Stephen J. Hill

**Affiliations:** ^1^ Division of Physiology, Pharmacology and Neuroscience, School of Life Sciences University of Nottingham Nottingham UK; ^2^ Centre of Membrane Proteins and Receptors (COMPARE) University of Birmingham and University of Nottingham Midlands UK; ^3^ Centre for Cancer Sciences, School of Medicine, Biodiscovery Institute University of Nottingham Nottingham UK; ^4^ Division of Biomolecular Science and Medicinal Chemistry, School of Pharmacy, Biodiscovery Institute University of Nottingham Nottingham UK; ^5^ Department of Oncology Nottingham University Hospitals NHS Trust UK

**Keywords:** adenosine A_2B_ receptor, antagonist, fluorescent ligand, ligand‐binding, macrophages, PSB603

## Abstract

Fluorescent ligands have proved to be powerful tools in the study of G protein‐coupled receptors in living cells. Here we have characterized a new fluorescent ligand PSB603‐BY630 that has high selectivity for the human adenosine A_2B_ receptor (A_2B_R). The A_2B_R appears to play an important role in regulating immune responses in the tumor microenvironment. Here we have used PSB603‐BY630 to monitor specific binding to A_2B_Rs in M1‐ and M2‐like macrophages derived from CD14+ human monocytes. PSB603‐BY630 bound with high affinity (18.3 nM) to nanoluciferase‐tagged A_2B_Rs stably expressed in HEK293G cells. The ligand exhibited very high selectivity for the A_2B_R with negligible specific‐binding detected at NLuc‐A_2A_R, NLuc‐A_1_R, or NLuc‐A_3_R receptors at concentrations up to 500 nM. Competition binding studies showed the expected pharmacology at A_2B_R with the A_2B_R‐selective ligands PSB603 and MRS‐1706 demonstrating potent inhibition of the specific binding of 50 nM PSB603‐BY630 to A_2B_R. Functional studies in HEK293G cells using Glosensor to monitor G_s_‐coupled cyclic AMP responses indicated that PSB603‐BY630 acted as a negative allosteric regular of the agonist responses to BAY 60–6583. Furthermore, flow cytometry analysis confirmed that PSB603‐BY630 could be used to selectively label endogenous A_2B_Rs expressed on human macrophages. This ligand should be an important addition to the library of fluorescent ligands which are selective for the different adenosine receptor subtypes, and will enable study of the role of A_2B_Rs on immune cells in the tumor microenvironment.

## Graphical abstract

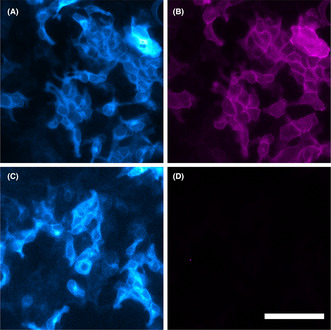

**Abbreviations**
BODIPY: boron‐dipyrromethene
cAMP: cyclic adenosine monophosphate
cDNA: complementary deoxyribonucleic acid
cryo‐EM: cryo‐electron microscopy
DMEM: Dulbecco's Modified Eagles Medium
EDTA: ethylenediaminetetraacetic acid
ELISA: enzyme linked immuno‐absorbent assay
FBS: fetal bovine serum
FCS: fetal calf serum
GMCSF: granulocyte‐macrophage colony‐stimulating factor
GPCR: G protein coupled receptor
HBSS: Hank's balanced salt solution
HEK293 cells: human embryonic kidney cells
HEPES: 4‐(2‐hydroxyethyl)‐1‐piperazineethanesulfonic acid
HPLC: high performance liquid chromatography
IFNγ: interferon γ
IL‐10: interleukin 10
IL‐12: interleukin 12
LC–MS: liquid chromatography‐mass spectrometry
LPS: lipopolysaccharides
MCSF: macrophage colony‐stimulating factor
NanoBRET: nanoluciferase bioluminescence resonance energy transfer
NECA: 5‐(N‐ethylcarboxamido) adenosine
Nluc: nanoluciferase
NMR: Nuclear Magnetic Resonance
PBMC: peripheral blood mononuclear cells
RP‐HPLC: reversed phase high performance liquid chromatography
S.E.M.: standard error of mean

## 1 INTRODUCTION

Adenosine acts via four different G protein coupled receptor (GPCR) subtypes (A_1_R, A_2A_R, A_2B_R and A_3_R).^1^, ^2^ A_1_R and A_3_R primarily couple to Gα_i/o_ proteins and inhibit adenylyl cyclase activity, whilst the A_2A_R preferentially couples to Gα_s_ proteins and stimulates the formation of cyclic AMP (cAMP).^1^, ^2^, ^3^, ^4^ In contrast, the A_2B_R appears to be more promiscuous and, as well as coupling to Gα_s_ proteins,^4^ there is evidence of coupling of A_2B_R to other G‐proteins, most notably Gα_q/11_, Gα_i_ and Gα_12/13_ proteins.^5^, ^6^, ^7^, ^8^ Interestingly, there are differences in the extent to which different A_2B_R agonists activate different signaling pathways. Thus, adenosine and NECA activate most members of the four Gα protein families (Gα_s_, Gα_q/11_, Gα_i_, and Gα_12/13_) whilst the A_2B_‐selective partial agonist BAY 60–6583^4^, ^5^ preferentially couples to Gα_s_, Gα_15_, and Gα_12_.^8^

Crystal and/or cryo‐electron microscopy (cryo‐EM) structures have now been reported for both the A_2A_R and A_2B_R. The crystal structure of the A_2A_R has been obtained in antagonist‐^9^ and agonist‐^10^, ^11^ bound conformations. A cryo‐EM structure is also available for the A_2A_R coupled to an engineered heterotrimeric G protein.^12^ The A_2B_R is closely related to the A_2A_R, but has low affinity for NECA and adenosine.^3^, ^13^ Recently, two A_2B_R cryo‐EM structures co‐bound to NECA (PDB: 7XY7) or BAY 60–6583 (PDB: 7XY6) in the presence of an engineered heterotrimeric Gs protein have been published.^14^ The overall structure of A_2B_R‐NECA‐G_s_ is very similar to that of A_2A_R‐NECA.^14^ The A_2B_R‐BAY60‐6583‐G_s_ structure, however, revealed an orthosteric binding pocket similar to that of NECA, but with a secondary binding pocket extending out from the orthosteric binding site where residues V250^6.51^ and N273^7.36^ appear to be key determinants of its selectivity for A_2B_R.^14^

Recent therapeutic interest in A_2A_R and A_2B_R has focussed on the role of these Gαs‐coupled adenosine receptors on immune cells in relation to cancer progression. For example, activation of A_2A_Rs on the surface of immune cells can suppress the normal adaptive immune response to the formation of tumors and facilitate cancer growth and tumor cell dissemination.^15^, ^16^, ^17^, ^18^ This has led to the development of specific A_2A_R antagonists to inhibit the immunosuppressive effects of A_2A_Rs in the tumor microenvironment.^19^ The A_2B_R also appears to have a similar role in regulating the immune response in the tumor microenvironment.^20^, ^21^, ^22^ Furthermore, A_2B_R‐selective antagonists have been evaluated in patients with non‐small cell lung cancer.^20^ In addition, tumor‐derived exosomes have been shown to promote angiogenesis via A_2B_R signaling.^23^ Thus, these exosomes promote the polarization of macrophages towards an M2‐like phenotype and enhance the secretion of angiogenic factors.^23^ A key requirement for future studies on the relative role of adenosine receptors in the tumor microenvironment is the need to be able to monitor the expression level of A_2A_R and A_2B_R on the surface of individual immune cells. In this context, recent advances in fluorescent ligand technologies have begun to allow the development of live‐cell and single cell ligand‐receptor binding assays.^24^, ^25^, ^26^, ^27^

We have recently described the development of a series of fluorescent antagonist probes for A_2A_R.^28^, ^29^ The first series were developed from the A_2A_R‐selective antagonist preladenant (SCH420814 ^30^) and exhibited high affinity and selectivity for A_2A_R which allowed clear visualization of the receptor location in single living cells using confocal imaging.^28^ In a separate strategy, we also designed a fluorescent antagonist based on ZM241385 that incorporated a linker between the pharmacophore and the sulfo‐cyanine5 fluorophore (Cy5) that facilitated covalent transfer of the fluorphore to the A_2A_R.^29^ This was then used to monitor binding to human macrophages endogenously expressing the A_2A_R.^29^ Successful high affinity and A_2B_‐selective fluorescent ligands have also been developed previously (e.g., PSB‐12105) using a green‐emitting BODIPY fluorophore attached to 8‐substituted xanthine derivatives.^31^ The aim of the present study was to develop a red‐emitting fluorescent antagonist that is selective for A_2B_R. Here we have based our fluorescent probe design on the A_2B_R‐selective antagonist PSB603 ^4^, ^31^, ^32^ and demonstrate that it can be used to selectively monitor binding to endogenous adenosine A_2B_R in human macrophages.

## 2 MATERIALS AND METHODS

### 2.1 Materials

2‐(2‐Furanyl)‐7‐(2‐phenylethyl)‐7*H*‐pyrazolo[4,3‐*e*][1,2,4]triazolo[1,5‐*c*]pyrimidin‐5‐amine (Scheme 58261) (Cat# 2270), 2‐[[6‐Amino‐3,5‐dicyano‐4‐[4‐(cyclopropylmethoxy)phenyl]‐2‐pyridinyl]thio]‐acetamide (BAY 60–6583) (Cat# 4472), 8‐[4‐[4‐(4‐Chlorophenzyl)piperazide‐1‐sulfonyl phenyl]]‐1‐propylxanthine (PSB 603) (Cat#3198), *N*‐(4‐Acetylphenyl)‐2‐[4‐(2, 3,6,7‐tetrahydro‐2,6‐dioxo‐1,3‐dipropyl‐1*H*‐purin‐8‐yl)phenoxy]acetamide (MRS 1706) (Cat# 1584), 2‐(2‐Furanyl)‐7‐[3‐(4‐methoxyphenyl)propyl]‐7*H*‐pyrazolo[4,3‐*e*][1,2,4]triazolo[1,5‐*c*]pyrimidin‐5‐amine (Scheme 442416) (Cat#2463), *trans*‐4‐[(2‐Phenyl‐7*H*‐pyrrolo[2,3‐*d*]pyrimidin‐4‐yl)amino]cyclohexanol (SLV 320) (Cat#3344) and *N*‐[9‐Chloro‐2‐(2‐furanyl)[1,2,4]‐triazolo[1,5‐*c*]quinazolin‐5‐yl]benzene acetamide (MRS 1220) (Cat#1217) were purchased from Tocris Bioscience (Bristol, UK). Dimethyl Sulfoxide (DMSO) (Cat#D5879), lipopolysaccharide (LPS) (Cat# L2654) and Bovine Serum Albumin (BSA) (Cat# A7030) were purchased from Sigma‐Aldrich (Gillingham, UK).The cAMP GloSensor™ Human Embryonic Kidney 293 (HEK293G) cell line, the Nano‐Glo® Luciferase Assay System and GloSensor™ cAMP reagent were purchased from Promega Corporation (Madison, WI, USA). The human IL‐10 DuoSet® (Cat# DY2178), ELISA kit and interferon‐γ (Cat# 285‐IF‐100/CF) were purchased from R&D Systems. The BD OptEIA™ human IL‐12 (p70; Cat# 555183) ELISA kit was obtained from BD Biosciences. FuGENE and furimazine were obtained from Promega Corporation (Wisconsin, USA). SNAP‐Surface® Alexa Fluor® 488 was obtained from New England Biolabs (Hitchin, UK). All other chemicals were from Sigma‐Aldrich (Missouri, USA). Nunc™ Lab‐tek™ chambered coverglass (155361) were obtained from Thermo Fisher Scientific (Paisley, UK). 96‐well white clear‐bottomed plates and 35 mm Cellview 4‐quadrant culture dishes were from Greiner bio‐one (Kremsmunster, Austria). The synthesis of the fluorescent ligands AV039 (compound 19 in^33^), EC069 (compound 44b in^34^) and EC005 (compound 12 in^22^) have been described previously.

### 2.2 Chemistry

**FIGURE 1:**
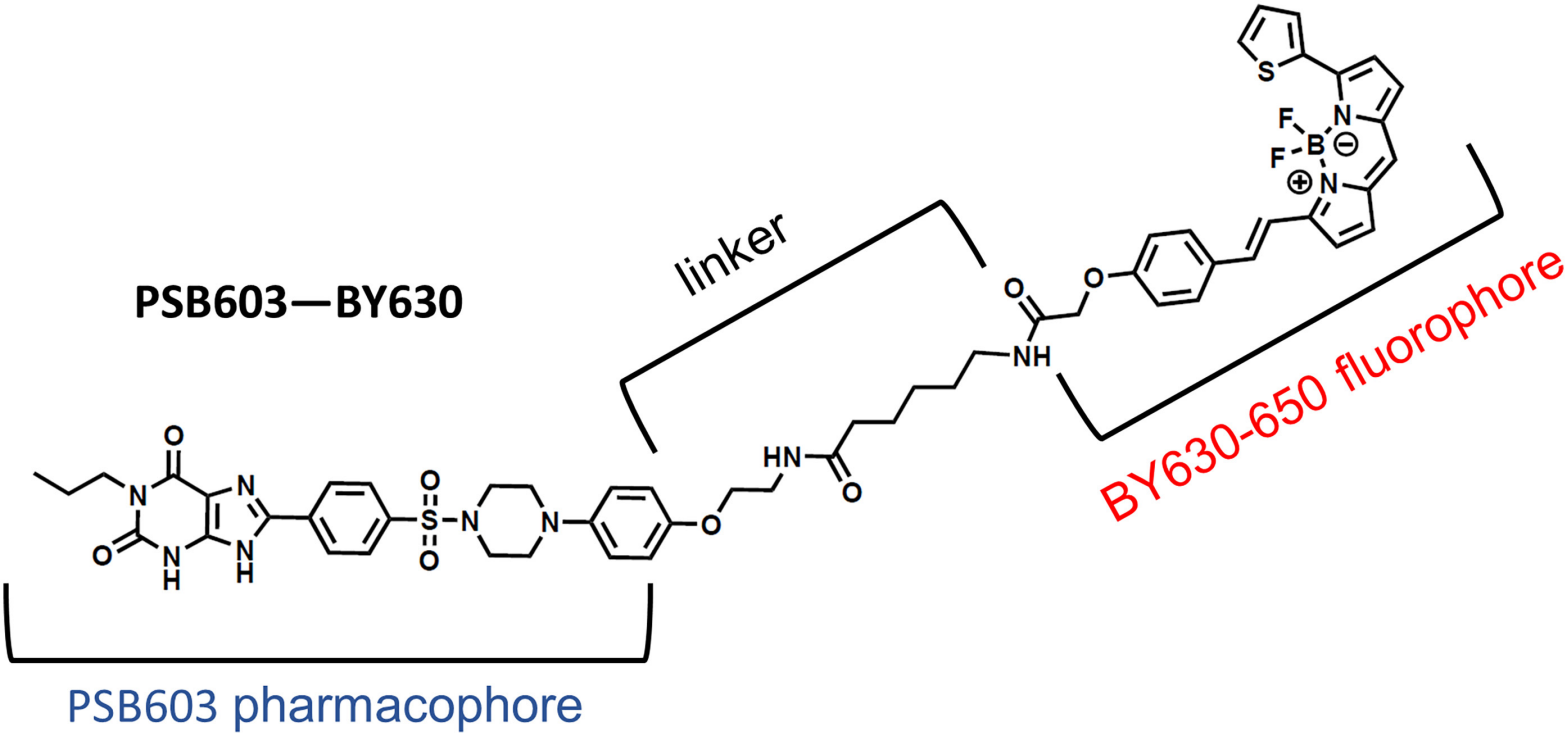
Structure of PSB603‐BY630.

Chemicals and solvents of analytical and HPLC grade were purchased from commercial suppliers and used without further purification. BODIPY‐630/650‐X‐SE was purchased from Molecular Probes (Thermo Fisher Scientific). All reactions were carried out at ambient temperature unless otherwise stated. Reactions were monitored by thin‐layer chromatography on commercially available silica pre‐coated aluminium‐backed plates (Merck Kieselgel 60 F254). Visualization was under UV light (254 nm and 366 nm), followed by staining with ninhydrin or KMnO_4_ dips. Flash column chromatography was performed using silica gel 60, 230–400 mesh particle size (Sigma Aldrich). NMR spectra were recorded on a Bruker‐AV 400. ^1^H spectra were recorded at 400.13 Hz and ^13^C NMR spectra at 101.62 Hz. All ^13^C NMR are ^1^H broadband decoupled. Solvents used for NMR analysis (reference peaks listed) were CDCl_3_ supplied by Cambridge Isotope Laboratories Inc., (δ_H_ = 7.26 ppm, δ_C_ = 77.16) and CD_3_OD supplied by VWR (δ_H_ = 3.31 ppm and δ_C_ = 49.00). Chemical shifts (δ) are recorded in parts per million (ppm) and coupling constants are recorded in Hz. The following abbreviations are used to described signal shapes and multiplicities; singlet (s), doublet (d), triplet (t), quadruplet (q), broad (br), dd (doublet of doublets), ddd (double doublet of doublets), dtd (double triplet of doublets) and multiplet (m). Spectra were assigned using appropriate COSY and HSQC experiments. Processing of the NMR data was carried out using the NMR software Topspin 3.0. LC–MS spectra were recorded on a Shimadzu UFLCXR system coupled to an Applied Biosystems API2000 and visualized at 254 nm (channel 1) and 220 nm (channel 2). LC–MS was carried out using a Phenomenex Gemini‐NX C18 110A, column (50 mm × 2 mm × 3 μm) at a flow rate 0.5 mL/min over a 5 min period. All high resolution mass spectra (HRMS) were recorded on a Bruker microTOF mass spectrometer using MS electrospray ionization operating in positive ion mode. RP‐HPLC was performed on a Waters 515 LC system and monitored using a Waters 996 photodiode array detector at wavelengths between 190 and 800 nm. Spectra were analyzed using Millenium 32 software. Semi‐preparative HPLC was performed using YMC‐Pack C8 column (150 mm × 10 mm × 5 μm) at a flow rate of 5.0 mL/min using a gradient method of 40%–95% B over 15 min (Solvent A = 0.01% formic acid in H_2_O, solvent B = 0.01% formic acid in CH_3_CN (method A)) or 40%–75% B over 10 min (Solvent A = 0.01% formic acid in H_2_O, solvent B = 0.01% formic acid in CH_3_CN (method B)). Analytical RP‐HPLC was performed using a YMC‐Pack C8 column (150 mm × 4.6 mm × 5 μm) at a flow rate of 1.0 mL/min. Final products were one single peak and >95% pure. The retention time of the final product is reported using a gradient method of 5%–95% solvent B in solvent A over 25 min. (Solvent A = 0.01%) formic acid in H_2_O, (solvent B = 0.01%) formic acid in CH_3_CN. Full experimental detail for the synthesis of PSB603‐BY630 (Figure 1) can be found in the Supplementary Information.

### 2.3 Cell lines

HEK293T cells were obtained from ATCC (Virginia, USA). A clonal HEK 293 cell line stably expressing the cAMP GloSensor (20F) biosensor (HEK293G)^4^, ^35^ was obtained from Promega Corporation (Madison, WI, USA). HEK293T and HEK293G cell lines were maintained in Dulbecco's Modified Eagles Medium (DMEM; Sigma‐Aldrich, Missouri USA) supplemented with 10% fetal bovine serum (FBS; Sigma‐Aldrich, Missouri USA) at 37°C 5% CO_2_. The generation of HEK293T or HEK293G cells stably expressing NanoLuc‐A_1_R, NanoLuc‐A_2B_R and NanoLuc‐A_3_R have been described previously.^28^, ^36^

### 2.4 Transient expression of NanoLuc‐A_2A_R

The human A_2A_ receptor cDNA was obtained from Missouri S&T cDNA Resource Centre (www.cdna.org) in a pcDNA3.1 expression vector. An N‐terminal nanoluciferase (NLuc)‐labeled human A_2A_R receptor constructs (NLuc‐A_2A_R) was then generated in frame with the full length NLuc incorporating a rat 5‐HT_3A_ membrane localisation signal sequence in pcDNA3.1 as described previously.^37^ For transient transfections, HEK293G cells were seeded at 20000 cells/well into white walled, clear bottomed 96‐wells plates (Greiner Bio‐One, Stonehouse, UK), coated with 10 μg/mL poly‐D‐lysine, in 100 μL medium/well and incubated at 37°C and 5% CO_2_ for 18–24 h. After 24 h, cells were transfected with 100 ng per well of pcDNA3.1 NLuc‐A_2A_R diluted in Opti‐MEM, using FuGENE HD at a 3:1 reagent to DNA ratio following manufacturer's instructions. Following a 10 min incubation at room temperature, 5 μL per well of transfection mix was added to each well. Cells were left at 37°C 5% CO_2_, for 24 h prior to NanoBRET assays.

### 2.5 NanoBRET binding assay

Saturation and competition binding assays were performed as previously described.^36^ Briefly, cells were seeded in 96‐well white clear‐bottomed Greiner plates pre‐treated with 10 μg/mL poly‐D‐lysine (Sigma‐Aldrich, Missouri USA) at a density of 30 000–35 000 cells per well in DMEM supplemented with 10% FBS. The following day medium was removed and cells were incubated with PSB603‐BY630 in the presence or absence of 1 μM MRS1706 (saturation binding assays) or competing ligand in the presence of 50 nM PSB603‐BY630 (competition binding assays) in HEPES buffered saline solution (HBSS; 145 mM NaCl, 5 mM KCl, 1.3 mM CaCl_2_, 1 mM MgSO_4_, 10 mM HEPES, 2 mM sodium pyruvate, 1.5 mM NaHCO_3_, 10 mM D‐glucose, pH 7.45) with 0.1% bovine serum albumin for 1 h at 37°C. The NanoLuc substrate, furimazine (Promega Corporation, Wisconsin, USA), was then added to each well (1:400 dilution) and the plate was incubated for 10 min in the dark at 37°C. The resulting bioluminescence resonance energy transfer (BRET) was measured using a PHERAstar FS plate reader (BMG Labtech) at 37°C. For each well, filtered light emissions at 460 nm (80 nm bandpass) and >610 nm (longpass) for the BODIPY630/650 ligand were simultaneously measured. BRET ratios were calculated by dividing the 610 nm emission by the 460 nm emission. All conditions were performed in 2–6 replicates within each plate. For kinetic experiments, cells were preincubated for 10 min with 1:400 dilution of furimazine prior to addition of 200 nM PSB603‐BY630 and BRET ratios determined every 0.5 min. At 60 min, 10 μM MRS‐1706 was added and the ligand‐binding kinetics followed for a further 60 min.

### 2.6 cAMP GloSensor™ luminescence assay

The cAMP GloSensor™ luminescence assay was performed according to the manufacturer's instructions (Promega Corporation, Madison, WI, USA). Briefly, after 24 h incubation at 37°C and 5% CO_2_ after cell plating (40 000 cells/well in 100 μL), medium was aspirated from each well of the 96‐well plate. Cells were incubated in 50 μL HEPES buffered saline solution (HBSS; 2 mM sodium pyruvate, 145 mM NaCl, 10 mM D‐glucose, 5 mM KCl, 1 mM MgSO_4_.7H_2_O, 10 mM HEPES, 1.3 mM CaCl_2_, 1.5 mM NaHCO_3_ in double‐distilled water, pH 7.45) containing 3% GloSensor™ cAMP reagent at 37°C for 1.5 h. For agonist studies, an initial baseline luminescence read was made at time zero, the plate was then removed from the plate‐reader and a further 50 μL HBSS containing agonist (2× final concentration) or HBSS (vehicle control) added. Luminescence was measured on an open channel (gain of 3600) immediately after these additions, and then continuously over 60 min, reading each well once every minute, by a PHERAstar FSX microplate reader (BMG Labtech, Offenburg, Germany) at 37°C. Increases in luminescence are indicative of intracellular cAMP accumulation, thus the temporal changes in relative cytosolic cAMP concentration were measured upon agonist or vehicle addition. Antagonist action was determined following 30 min pre‐incubation of HBSS in the presence of 20 and 200 nM PSB603‐BY630 and read as above. All conditions were performed in triplicates within each plate.

### 2.7 NanoBRET imaging

Cells were seeded onto 35 mm Cellview 4‐quadrant culture dishes (Greiner Bio‐one), which have a 10 mm glass coverslip bottom, in DMEM supplemented with 10% FBS at a density of 100 000 cells per quadrant 2 days prior to experiment in total volume of 500 μL. On the day of the experiment medium was replaced with HBSS in the presence or absence of PSB603‐BY630 (100 nM) and/or MRS1706 (10 μM) and incubated for 30 min at 37°C before imaging. Bioluminescence and NanoBRET imaging were performed on an Olympus LuminoView 200 microscope with a 60× NA1.42 oil immersion objective with a 0.5× tube lens, following addition of furimazine (1:800 dilution) (Promega). Images were captured by a C9100‐23B IMAGE EMX2 camera (Hamamatsu, Japan) with gain set at 200 for all channels. Filtered bioluminescence was captured using a 438/24 bandpass filter, BRET in the presence of PSB603‐BY630 was captured using a 650/50 nm bandpass filter. For the NLuc‐A_2B_R stable cell line exposure times were set at 10 s for filtered bioluminescence and 75 sec for BRET. Raw intensity values were determined for three regions of interest per experiment per condition and the BRET ratio calculated by dividing the raw intensity recorded from the BRET capture by the filtered bioluminescence capture. Corrected BRET ratios were determined by subtracting the BRET ratio determined from a control quadrant (HBSS alone). For each condition five separate experiments were performed.

### 2.8 Human macrophage generation

One hundred and fifty milliliter peripheral blood was obtained in heparinised 60 mL syringes by venepuncture from healthy volunteers after written informed consent (Ethics from University of Nottingham Ethics committee, ref 161–1711). Peripheral blood mononuclear cells (PBMC) were immediately separated by density centrifugation over Lymphoprep (Stemcell, UK) at 800*g* for 25 min on low brake followed by washes in endotoxin‐free phosphate‐buffered saline (PBS, Sigma). PBMC were washed in MACS buffer (PBS + 1% fetal calf serum (FCS, Sigma) + 2 μM EDTA (Sigma)) then incubated with CD14 microbeads (Miltenyi Biotech) and monocytes isolated by magnetic separation on an AutoMACS Pro cell separator (Miltenyi). Cell purity was routinely assessed by flow cytometry (>95%). Purified CD14+ monocytes were differentiated into macrophages at 37°C/5% CO_2_ for 7 days at 1 × 10^6^/well in low‐attachment 24‐well plates (Corning Costar) in 1 mL macrophage medium (RPMI 1640 (Sigma) supplemented with 10% endotoxin‐free FCS (Sigma) and 1% sodium pyruvate (Sigma)) plus cytokines. For M1‐like macrophages, Granulocyte‐macrophage colony‐stimulating factor (GMCSF, Peprotech) was added at day 0 at 20 U/mL and for M2‐like macrophages, Macrophage colony‐stimulating factor (MCSF, Immunotools) at 10 ng/mL. Culture medium was supplemented at day 4 with equal volume of medium + GMCSF or MCSF as appropriate. Macrophage phenotype validation was confirmed based on morphological observation using a Nikon ECLIPSE TS100 inverted microscope in 20X magnification and by cytokine secretion profile with M1‐like macrophages secreting high IL‐12 and low IL‐10, and M2‐like macrophages secreting low IL‐12 and high IL‐10.

### 2.9 ELISA analysis of IL‐12 and IL‐10 secretion from M1‐ and M2‐ like human macrophages in response to stimulation as phenotypic confirmation

Following differentiation of CD14+ monocytes for 7 days with either GMCSF (for M1‐like macrophages) or MCSF (for M2‐like macrophages) as described above, M1‐ and M2‐like macrophages were dislodged from plates by incubation on ice for 25 min in cold endotoxin‐free PBS. Harvested macrophages were used for labelling and flow cytometry (see 2.10) and separately seeded at a density 5 × 10^4^ cells/well in a total volume of 100 μL of macrophage medium in a standard 96 well plate (ThermoFisher) for cytokine stimulation as a phenotypic readout. After resting for 2 h at 37°C/5% CO_2_, 100 μL of medium containing either LPS (1 mg/mL) plus interferon‐γ (IFNγ; 1000 U/mL) for M1‐macrophages or LPS (1 mg/mL) alone for M2‐like macrophages was added. Supernatants were collected from triplicate wells after a 24 h incubation at 37°C/5% CO_2_. A single well of unstimulated cells was run as negative control per macrophage type. The levels of IL‐10 or IL‐12 cytokines were determined using the Human IL‐10 DuoSet® or the BD OptEIA™ Human IL‐12 (p70) ELISA kits respectively according to manufacturers' instructions.

### 2.10 Macrophage labelling and flow cytometry

Harvested macrophages were resuspended in staining buffer (HBSS [Sigma] supplemented with 2.5% v/v FCS and Ethylenediaminetetraacetic acid‐ EDTA 5 mM) at 2 × 10^6^ cells/mL. Samples of 2 × 10^5^ cells in 200 μL were incubated with 100 nM PSB603‐BY630 for 20 min at RT with or without a 30 min RT pre‐incubation with 10 μM PSB603. BODIPY 630/650 fluorescence was measured on a MACSQuant 10 Flow Cytometer (Miltenyi) immediately after incubation (>5 × 10^4^ events acquired). Flow cytometry data were analyzed using FlowJo software v10. The gating strategy used for the flow cytometry experiments is provided in the Supplementary Information.

### 2.11 Data analysis

Data were analyzed using Prism 7.4 software (GraphPad, San Diego, USA). Saturation NanoBRET curves were fitted simultaneously for total (PSB603‐BY630) and non‐specific binding (in the presence of 10 μM MRS1706) using the following equation:

$$\text{Total binding}=\frac{B_{\max}\times \left[B\right]}{\left[B\right]+K_{D}}+m\times B+c$$

where *B*_max_ is the maximal specific binding, [*B*] is the concentration of the fluorescent ligand (nM), *K*_*D*_ is the equilibrium dissociation constant (nM), *m* is the slope of the non‐specific binding component, and *C* is the y‐axis intercept.

The affinities of ligands at the NLuc‐A_2A_R were calculated from competition binding data with a one‐site sigmoidal response curve given by the following equation:

$$\begin{array}{c} \%\text{Inhibition of specific binding}=\frac{\left(100\times \left[A^{n}\right]\right)}{\left(\left[A^{n}\right]+{\mathrm{IC}}_{50}^{n}\right)} \end{array}$$

where [A] is the concentration of unlabelled ligand, *n* is the Hill coefficient, and *IC*_50_ is the concentration of ligand required to inhibit 50% of fluorescent ligand. The *IC*_50_ values were then used to calculate the *K*_*i*_ values using the Cheng‐Prussoff equation:

$$K_{i}=\frac{{\mathrm{IC}}_{50}}{1+\frac{\left[L\right]}{K_{D}}}$$

where [*L*] is the concentration of PSB603‐BY630 in nM, and *K*_*D*_ is the dissociation constant of that fluorescent ligand in nM.

Bioluminescence and NanoBRET images were analyzed using ImageJ (http://rsb.info.nih.gov/ij; NIH, USA) and the Time Series Analyzer version 3.0 (https://imagej.nih.gov/ij/plugins/time‐series.html).^38^

For kinetic binding experiments, the BRET ratio obtained in the absence of fluorescent ligand was determined for each time point and subtracted from the total binding to obtain baseline‐corrected values for total binding at each time point. 60 min after addition of 200 nM PSB603‐BY630, 10 μM MRS‐1706 was added and the dissociation data fitted to the following equation to obtain values for the dissociation rate constant (*k*_off_) in min^−1^:

$$\text{Y}=\left(\mathrm{Yo}-\mathrm{NS}\right).e^{-k_{\mathrm{off}}.t}+\mathrm{NS}$$

where [Yo] is the binding at time 60 min (when 10 μM MRS‐1706 was added), NS is the non‐specific binding at infinite time, *k*_off_ is dissociation rate constant. The residence time in min was then calculated as the reciprocal of *k*_off_.

### 2.12 Nomenclature of targets and ligands

Key protein targets and ligands in this article are hyperlinked to corresponding entries in http://www.guidetopharmacology.org, the common portal for data from the IUPHAR/BPS Guide to PHARMACOLOGY,^39^ and are permanently archived in the Concise Guide to PHARMACOLOGY 2019/20.^40^

## 3 RESULTS

### 3.1 Synthesis of PSB603‐BY630

Development of the fluorescent ligand PSB603‐BY630 (Figure 1) was based on the A_2B_R selective xanthine‐based ligand PSB‐603 which displays sub‐nanomolar affinity for A_2B_R and has a large selectivity over the other adenosine receptor family subtypes (A_1_, A_2A_ and A_3_).^4^, ^32^ A fluorescent ligand generally consists of a targeting binding moiety, linker and fluorophore (Figure 1), and it is essential to take into account the properties of each of these components as each of them can affect the overall affinity and selectivity of the final fluorescent ligand.^41^, ^42^ BODIPY 630/650‐X was selected as the fluorophore moiety, due to its excellent optical properties and established use in the development of a toolbox of subtype‐selective fluorescent ligands for the family of the adenosine receptors.^28^, ^33^, ^34^ A previous structure–activity relationship (SAR) study of PSB‐603 analogues indicated that para‐substitution of the terminal aromatic ring, with lipophilic substituents, is well tolerated.^43^ Correspondingly, we sought to extend from this position via an aminoethyl handle which enabled us to directly attach the BODIPY 630/650‐X fluorophore (Figure 1). The synthesis of this fluorescent ligand is detailed in Supplementary Information (Figure S1).

### 3.2 Pharmacological characterization of PSB603‐BY630 binding to A_2B_R

**FIGURE 2:**
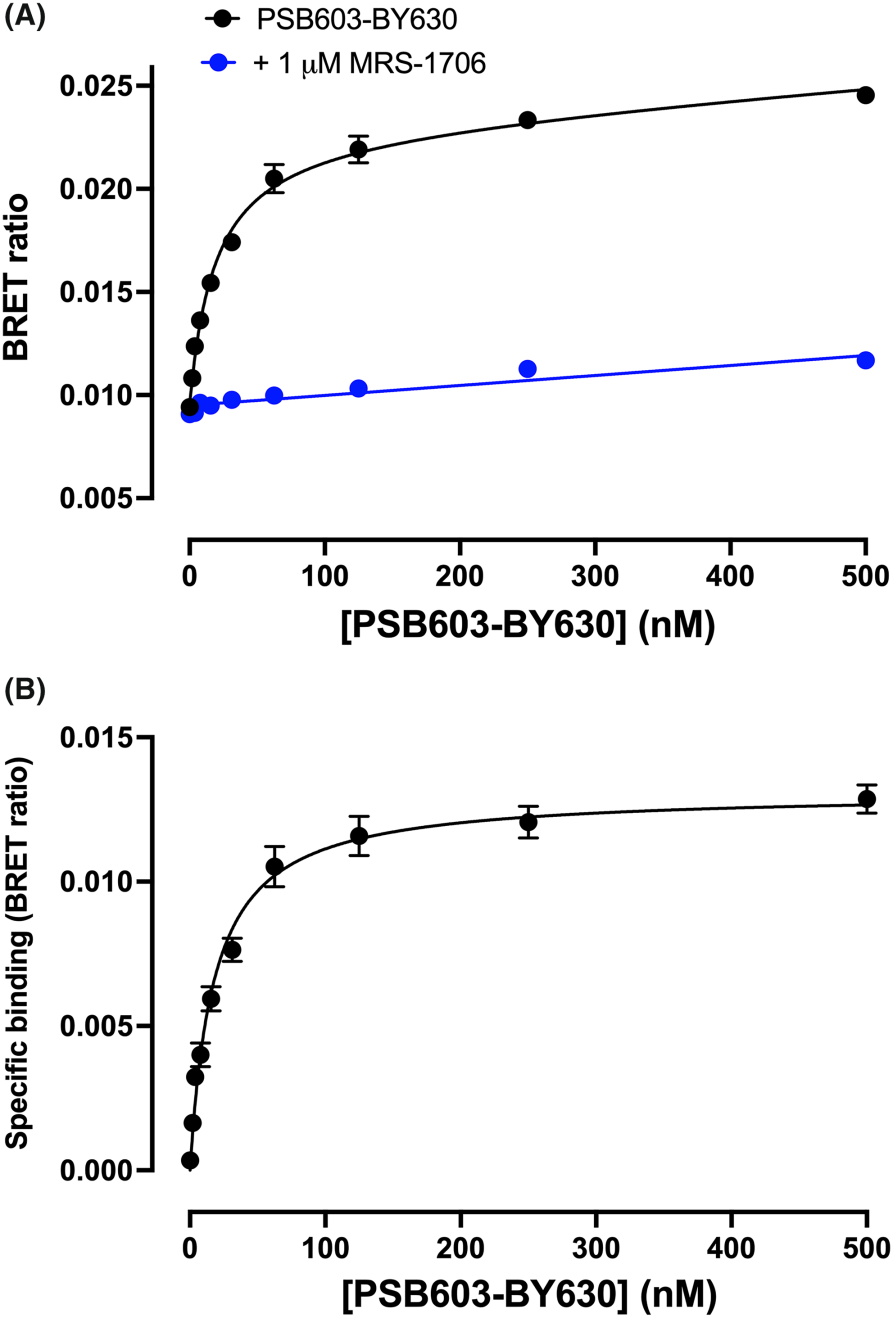
NanoBRET binding curves for PSB603‐BY630 in HEK293G cells exogenously expressing NLuc‐A_2B_R. (A) Cells were incubated with increasing concentrations of PSB603‐BY630 in the absence or presence of 1 μM MRS‐1706. (B) Specific‐binding of PSB603‐BY630 to NLuc‐A_2B_Rs. Data are mean ± S.E.M obtained in five independent experiments (each conducted in triplicate). The mean *K*_*D*_ value obtained in five separate experiments was 18.32 ± 1.65 nM.

An initial assessment of the binding of PSB603‐BY630 to human A_2B_Rs was made using NanoBRET in live HEK293G cells stably expressing A_2B_Rs tagged with an N‐terminal nanoluciferase (NLuc‐A_2B_R) (Figure 2). Clear saturable binding was detected at concentrations up to 500 nM that was prevented by simultaneous incubation with the A_2B_R‐selective inverse agonist MRS‐1706 (1 μM; Figure 2A). The mean *KD* value determined in five separate experiments for the specific component of binding (Figure 2B) was 18.32 ± 1.65 nM. This value was of a similar magnitude to that (3.6 nM) determined for [^3^H]‐PSB603 binding to membranes from CHO cells expressing the human A_2B_R.^13^

**FIGURE 3:**
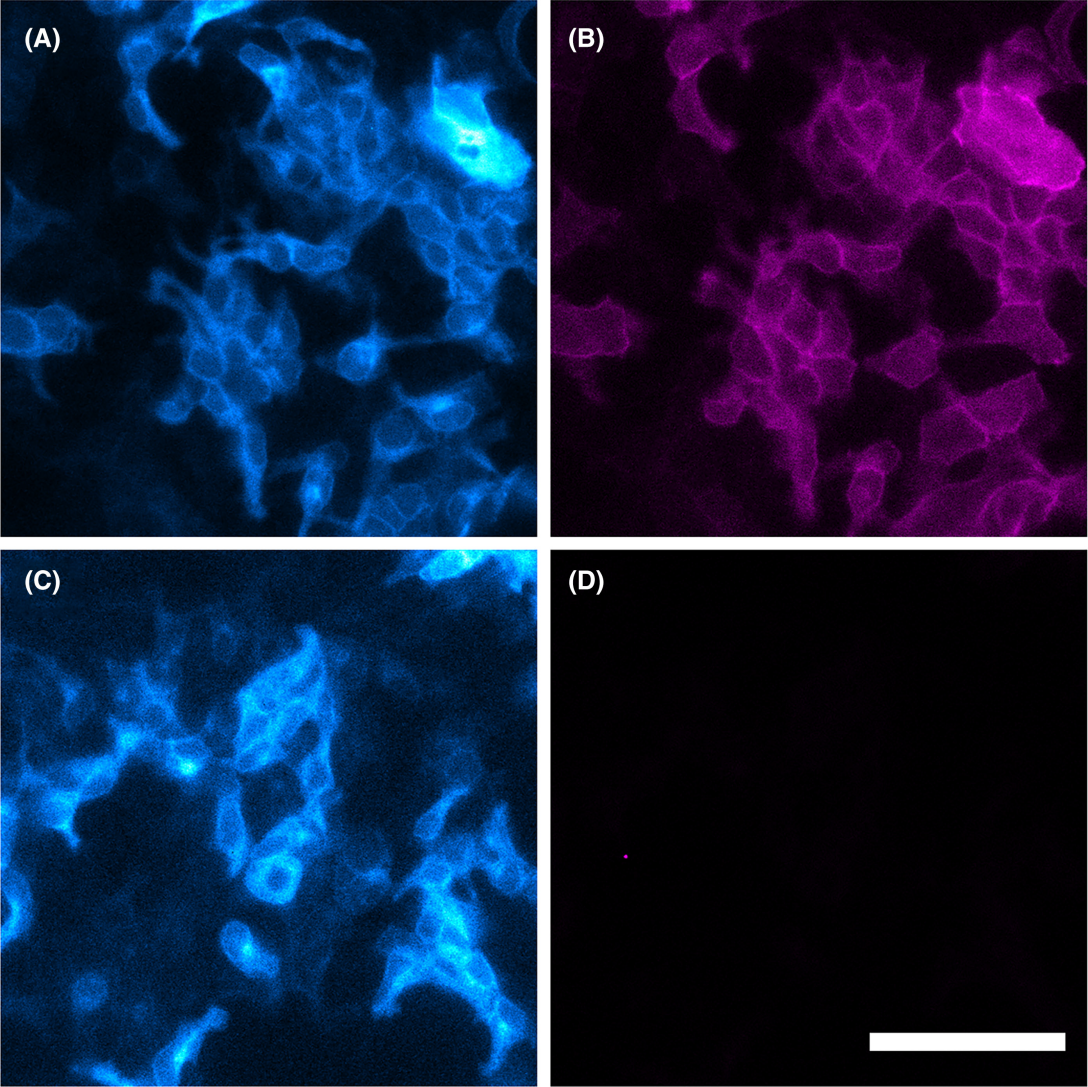
NanoBRET imaging of PSB603‐BY630 binding to HEK293G cells expressing NLuc‐A_2B_R. Cells were incubated with 100 nM PSB603‐BY630 in the absence (A, B) or presence (C, D) of 10 μM MRS‐1706 before addition of furimazine (1:800 dilution) and imaging. Filtered bioluminescence was captured using a 438/24 bandpass filter (cyan A & C). BRET was captured using a 650/50 nm bandpass filter (magenta B & D). Images are representative of those obtained in five independent experiments. Scale bar represents 100 μm.

Plate reader‐based saturable binding of PSB603‐BY630 monitored using NanoBRET in cell populations does not give, however, any indication of the subcellular location of the ligand‐receptor interaction in intact cells. To gain some insight into cellular location we also monitored the binding of PSB603‐BY630 to membrane‐bound A_2B_Rs in individual cells using bioluminescence imaging (Figure 3). In these experiments cells were incubated with 100 nM PSB603‐BY630 in the absence and presence of 10 μM MRS‐1706 for 30 min before addition of furimazine (1:800 dilution) and subsequent imaging. Filtered bioluminescence was captured for 10 s using a 438/24 bandpass filter in order to detect the location of the nanoluciferase‐tagged A_2B_Rs (cyan in Figure 3A,C). It is clear that there is substantial expression of the NLuc‐A_2B_R at the cell surface. A longer integration time (75 s) was used to monitor the ligand‐binding BRET signal using a 650/50 nm bandpass filter (magenta in Figure 3B,D). This showed clear binding to cell surface receptors that can be completely prevented by co‐incubation with the A_2B_R‐selective inverse agonist MRS‐1706 (Figure 3B,D).

**FIGURE 4:**
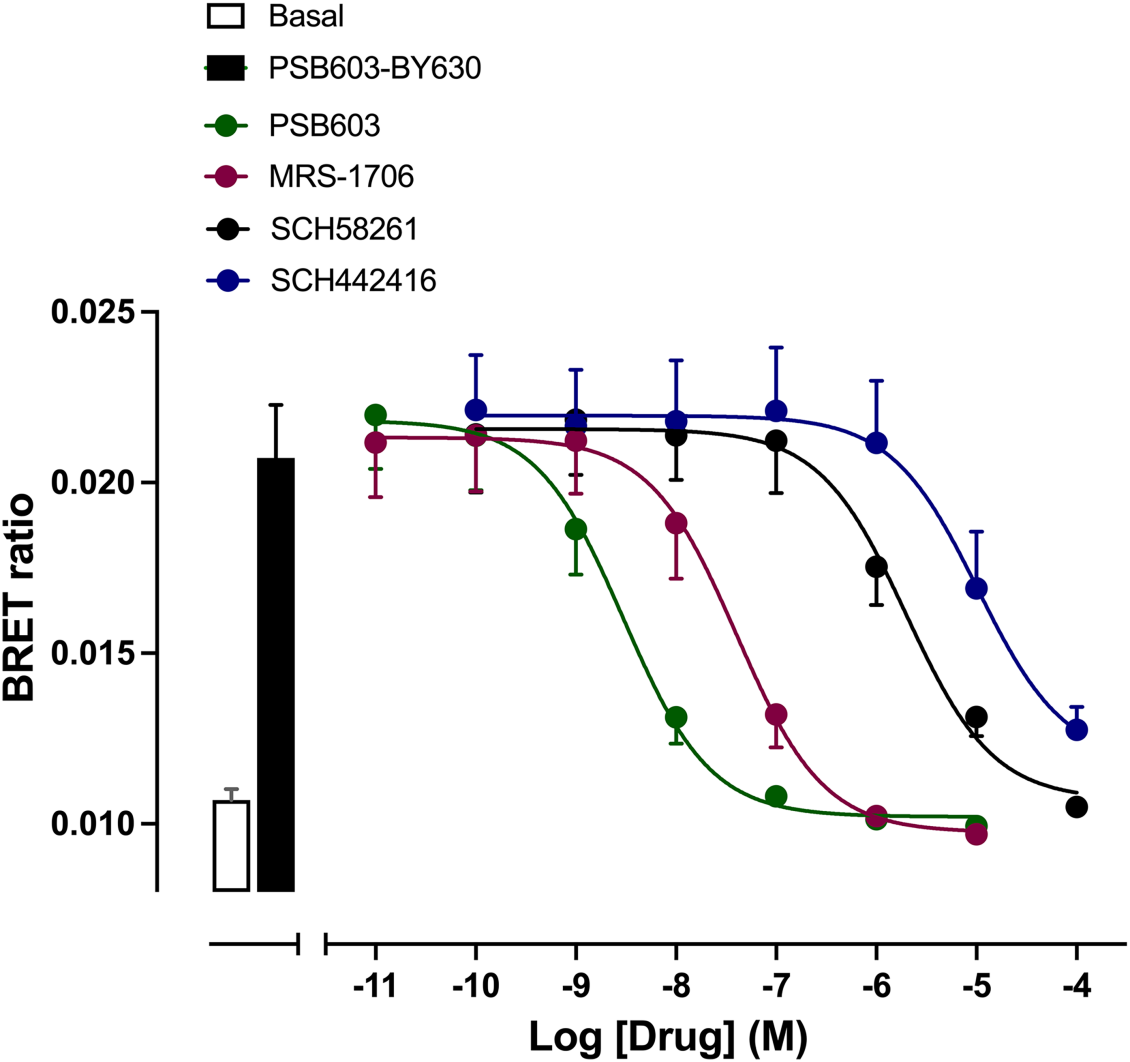
NanoBRET competition binding in HEK293G cells exogenously expressing NLuc‐A_2B_R. Cells were incubated with 50 nM PSB603‐BY630 in the absence or presence of competing ligands Data are mean ± S.E.M. from five independent experiments. The open and closed bars show the BRET ratio obtained in the absence and presence of 50 nM PSB603‐BY630 respectively.

Competition binding experiments demonstrated that the binding of 50 nM PSB603‐BY630 could be inhibited by a panel of different adenosine receptor‐selective ligands (Figure 4; Table 1) with an appropriate pharmacology for binding selectively to the A_2B_R. The most potent inhibitors were the A_2B_R antagonist PSB603^4^, ^32^ and the A_2B_R selective inverse agonist MRS‐1706.^44^ In contrast, the selective A_2A_R‐antagonists SCH442416 and SCH58261,^45^, ^46^ the A_1_R‐selective antagonist SLV320^34^ and the A_3_R‐selective antagonist MSR1220^32^ were much weaker (Figure 4; Table 1).

**TABLE 1:** Log IC_50_ and apparent log *K*_*i*_ values for inhibition of the binding of 50 nM PSB603‐BY630 obtained in five separate experiments.

Competitor	Log IC_50_	Appararent Log *K*_*i*_	*n*
PSB603	−8.55 ± 0.08	−9.12 ± 0.08	5
MRS1706	−7.44 ± 0.09	−8.01 ± 0.09	5
SCH442416	−5.02 ± 0.14	−5.59 ± 0.14	5
SCH58261	−5.68 ± 0.09	−6.26 ± 0.09	5
SLV320	−5.19 ± 0.18	−5.76 ± 0.18	5
MRS1220	−5.82 ± 0.19	−6.40 ± 0.19	5

*Note*: Values show mean ± S.E.M. Apparent log *K*_*i*_ values were calculated from IC_50_ values on the assumption that there is a competition between the inhibitor and PSB603‐BY630 for the same binding site.

**FIGURE 5:**
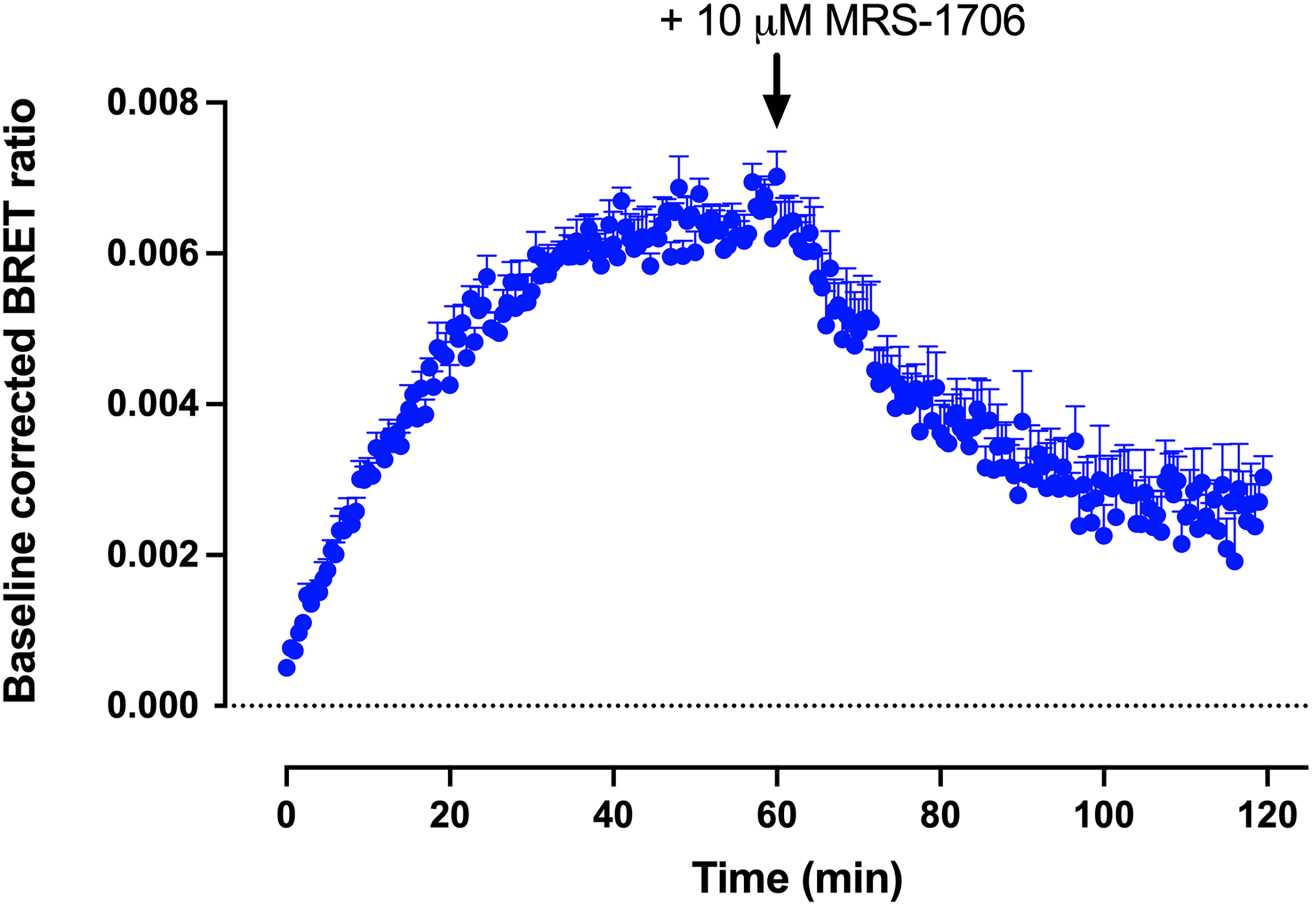
Ligand‐binding kinetics of 200 nM PSB603‐BY630 in HEK293G cells exogenously expressing NLuc‐A_2B_R. BRET ratios for the total binding of 200 nM PSB603‐BY630 were obtained every 30 sec. In parallel, data were also collected in the absence of fluorescent ligand for each time point and these data were subtracted from the total binding to obtain baseline‐corrected values for total binding at each time point. Sixty minutes after addition of 200 nM PSB603‐BY630, 10 μM MRS‐1706 was added to initial dissociation of the fluorescent ligand. Values show mean ± S.E.M of quadruplicate determinations in a single representative experiment. Similar data were obtained in four other experiments. The data points for the dissociation phase of the experiment were then fitted to a single exponential function to determine the the dissociation rate constant (*k*_off_) in min^−1^ as described under Methods. In this representative experiment the calculated *K*_off_ value was 0.056 min^−1^. The mean *K*_off_ value obtained in the five repeat experiments was 0.065 ± 0.003 min^‐1^.

Ligand‐binding kinetics of 200 nM PSB603‐BY630 indicated that equilibrium was achieved within 60 min at 37°C (Figure 5). At 60 min, 10 μM MRS‐1706 was then added to initiate fluorescent ligand dissociation (Figure 5). Fitting a single exponential function to these data allowed the dissociation rate constant *k*_off_ to be determined. This yielded a mean value of 0.065 ± 0.003 min^−1^ for *k*_off_ in 5 independent experiments. This equates to an average residence time of the fluorescent ligand of 15.4 min.

### 3.3 Receptor selectivity of PSB603‐BY630

**FIGURE 6:**
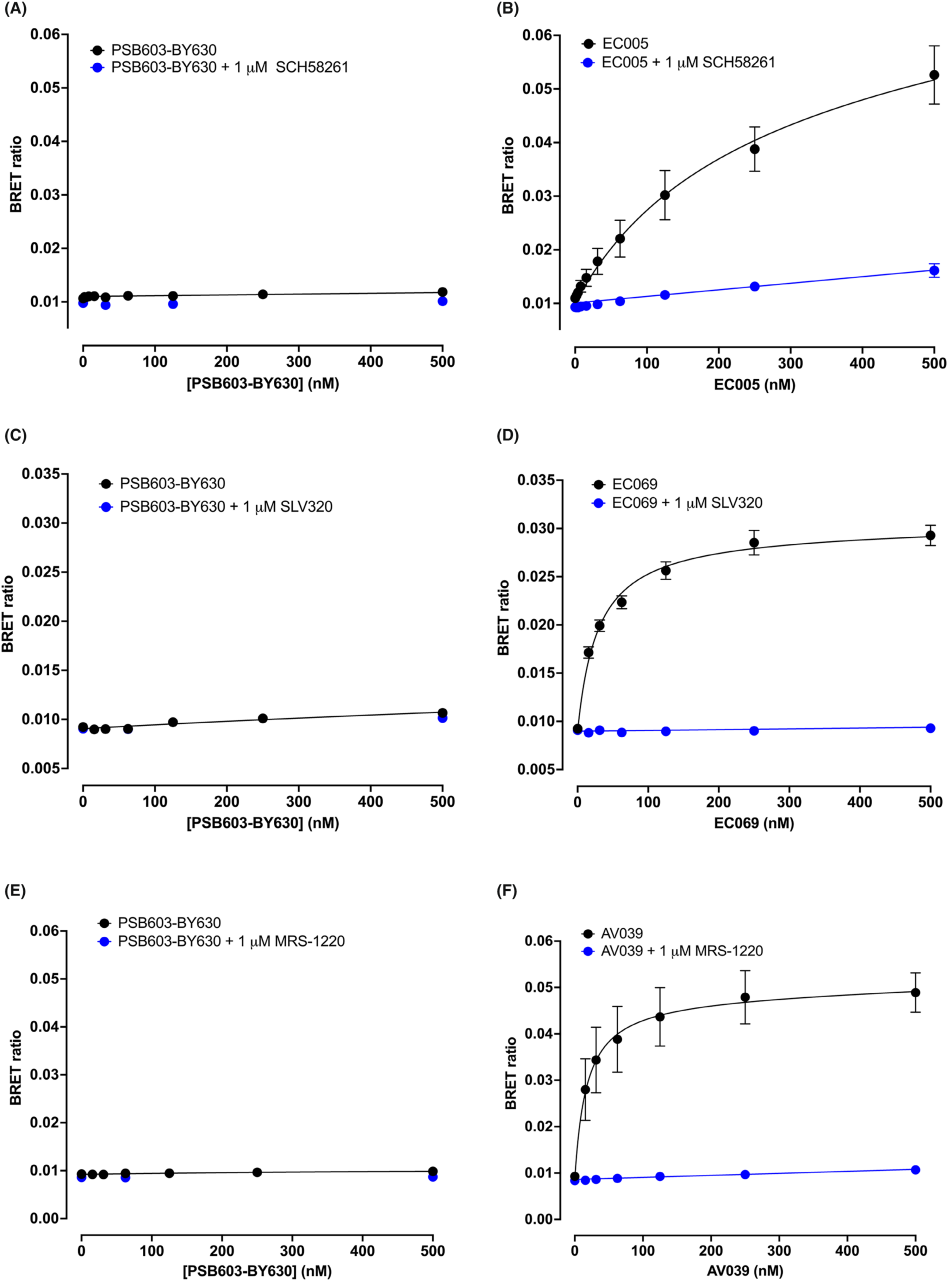
NanoBRET binding curves for PSB603‐BY630 and receptor‐selective fluorescent ligands binding to NLuc‐tagged A_1_, A_2A_ or A_3_ adenosine receptors. (A, B) Total and non‐specific binding of (A) PSB603‐BY630 or (B) EC‐005 to transiently transfected NLuc‐A_2A_R obtained in the absence and presence of 1 μM of the A_2A_R‐selective antagonist SCH58261. Data are mean ± S.E.M obtained in five independent experiments (each conducted in duplicate). (C, D) Total and non‐specific binding of (C) PSB603‐BY630 or (D) EC‐069 to NLuc‐A_1_R in a stable HEK293T cell line obtained in the absence and presence of 1 μM of the A_1_R‐selective antagonist SLV320. Data are mean ± S.E.M obtained in five independent experiments (each conducted in triplicate). (E, F) Total and non‐specific binding of (E) PSB603‐BY630 or (F) AV‐039 to NLuc‐A_3_R in a stable HEK293G cell line obtained in the absence and presence of 1 μM of the A_3_R‐selective antagonist MRS‐1220. Data are mean ± S.E.M obtained in five independent experiments (each conducted in triplicate).

To investigate the receptor selectivity of PS603‐BY630, we undertook saturation binding experiments in HEK293G cells transiently transfected with the human NLuc‐A_2A_R (Figure 6A), a stable HEK293T cell line expressing the human NLuc‐A_1_R (Figure 6C) or a stable HEK293G cell expressing the human NLuc‐A_3_R (Figure 6E). At concentrations up to 500 nM, PSB603‐BY630 showed negligible specific binding to NLuc‐A_2A_R, NLuc‐A_1_R or NLuc‐A_3_R (Figure 6). In marked contrast, high affinity specific binding was detected in parallel experiments on each receptor with receptor‐selective fluorescent ligands for A_2A_R (EC005^22^; Figure 6B), A_1_R (EC069^34^; Figure 6D) and A_3_R (AV039^33^; Figure 6F), respectively.

### 3.4 Functional cAMP responses in HEK293G cells endogenously expressing A_2B_Rs

**FIGURE 7:**
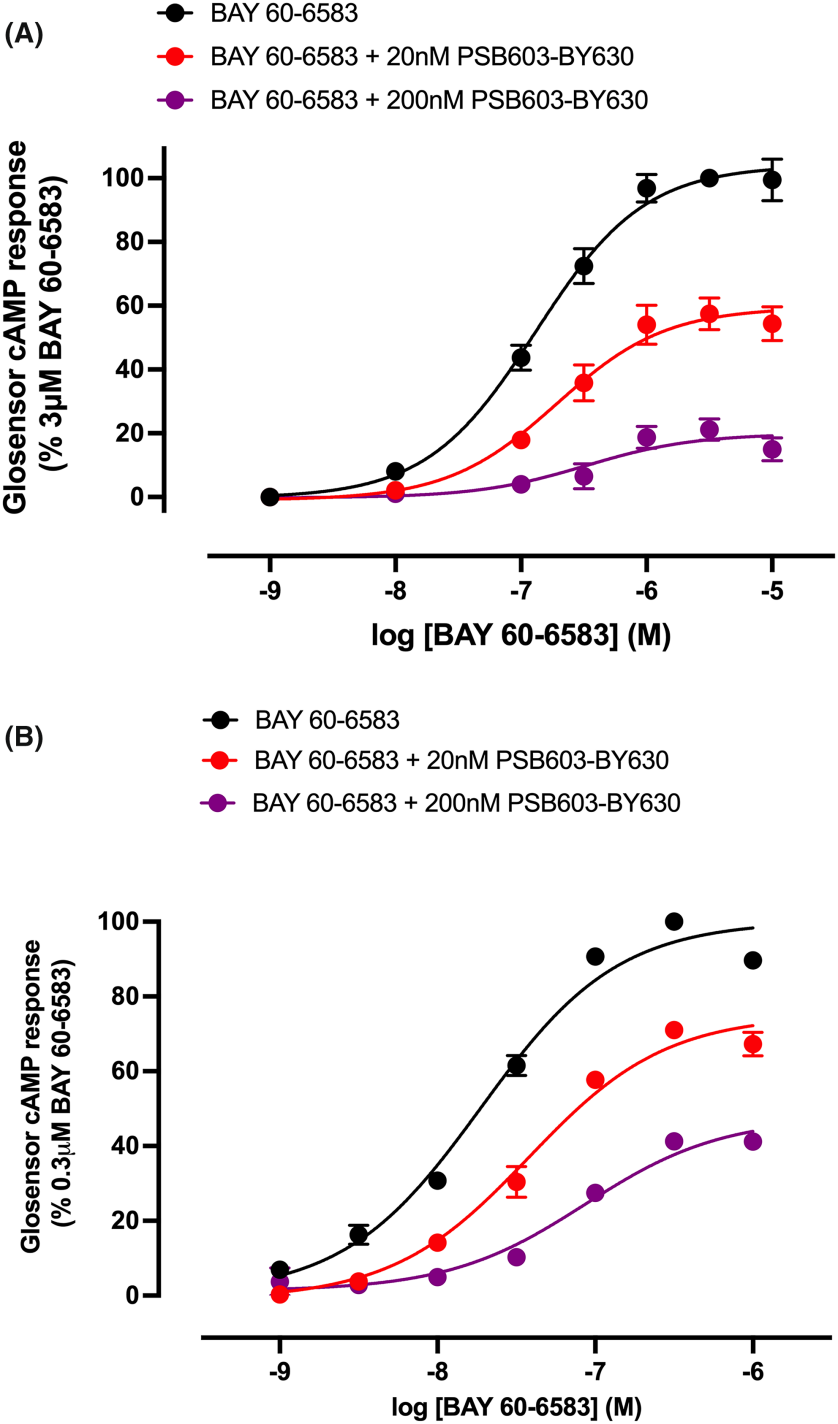
Effect of PSB603‐BY630 on Glosensor cAMP concentration‐response curves to the A_2B_‐selective agonist BAY 60–6583 in (A) HEK293G cells endogenously expressing A_2B_R or (B) HEK293G cells overexpressing NLuc‐A_2B_R. Concentration response curves were obtained in the absence and presence of 20 nM or 200 nM PSB603‐BY630. Values are mean ± S.E.M. of five separate experiments carried out in triplicate. Data represent peak luminescence response and are expressed as a percentage of the peak luminescence response to 3 μM BAY 60–6583 (in a) or 0.3 μM BAY 60–6583 (in b) obtained in the absence of antagonist in each individual experiment.

HEK293G cells that express the cAMP biosensor Glosensor also endogenously express both A_2B_R and A_2A_R.^4^ These cells therefore provide an opportunity to evaluate the pharmacological characteristics of PSB603‐BY630 in cells that express endogenous and untagged A_2B_Rs. We have previously shown that the A_2B_‐selective agonist BAY 60–6583 can elicit selective A_2B_R‐mediated Glosensor responses in these cells.^4^ Here we show that 20 nM and 200 nM PSB603‐BY630 produces a marked and significant (*p* <.01 and *p* <.0001 respectively; two way ANOVA) concentration‐dependent reduction in the maximal response to BAY 60–6583 without altering the EC_50_ of the A_2B_R agonist (Figure 7A; Table 2). These data are very similar to those reported previously for non‐fluorescent PSB603.^4^ Furthermore, in a stable cell line overexpressing human NLuc‐A_2B_Rs, the EC_50_ values for BAY 60–6583 were shifted to lower agonist concentrations consistent with an increase in the spare receptor reserve caused by A_2B_R overexpression (Figure 7B; Table 2). In these cells 20 nM and 200 nM PSB603‐BY630 produced a small increase in the EC_50_ for BAY 60–6583 (Table 2) that was accompanied by a significant decrease in the maximal response to BAY 60–6583 (p < 0.001 and p < 0.0001 respectively; two way ANOVA; Figure 7B; Table 2).

**TABLE 2:** Log EC_50_ and E_MAX_ values obtained in HEK293G cells endogenously expressing A_2B_R or HEK293G cells expressing recombinant human A_2B_R for BAY 60–6593 obtained in the absence and presence of increasing concentrations of PSB603BY630.

Agonist treatment	Endogenous HEK293G Log EC_50_	Endogenous HEK293G E_MAX_ (% of response to 3 μM BAY‐60‐6583)	*n*	HEK293G A_2B_AR Log EC_50_	HEK293G A_2B_AR E_MAX_ (% of response to 0.3 μM BAY‐60‐6583)	*n*
BY 60–6583	−6.89 ± 0.06	104.15 ± 3.09	5	−7.73 ± 0.03	100.2 ± 1.29	5
BY 60–6583 + 20 nM PSB603‐BY630	−6.71 ± 0.10	59.98 ± 5.76**	5	−7.43 ± 0.07*	75.13 ± 2.48***	5
BY 60–6583 + 200 nM PSB603‐BY630	−6.51 ± 0.16	20.63 ± 3.54****	5	−7.06 ± 0.03***	47.67 ± 0.86****	5

*Note*: E_MAX_ values are expressed as a percentage of the response obtained with 3 μM BAY 60–6583 or 0.3 μM BAY 60–6583 in cells recombinant expressing A_2B_R. Significant differences to that seen in the absence of antagonist are indicated (*p <.05, ** *p* <.01, *** *p* <.001 or ****p <.0001, 2‐way ANOVA with Dunnett’s multiple comparison test). Data are expressed as mean ± S.E.M. of 5 separate experiments.

### 3.5 PSB603‐BY630 binding to human M1‐like and M2‐like macrophages

**FIGURE 8:**
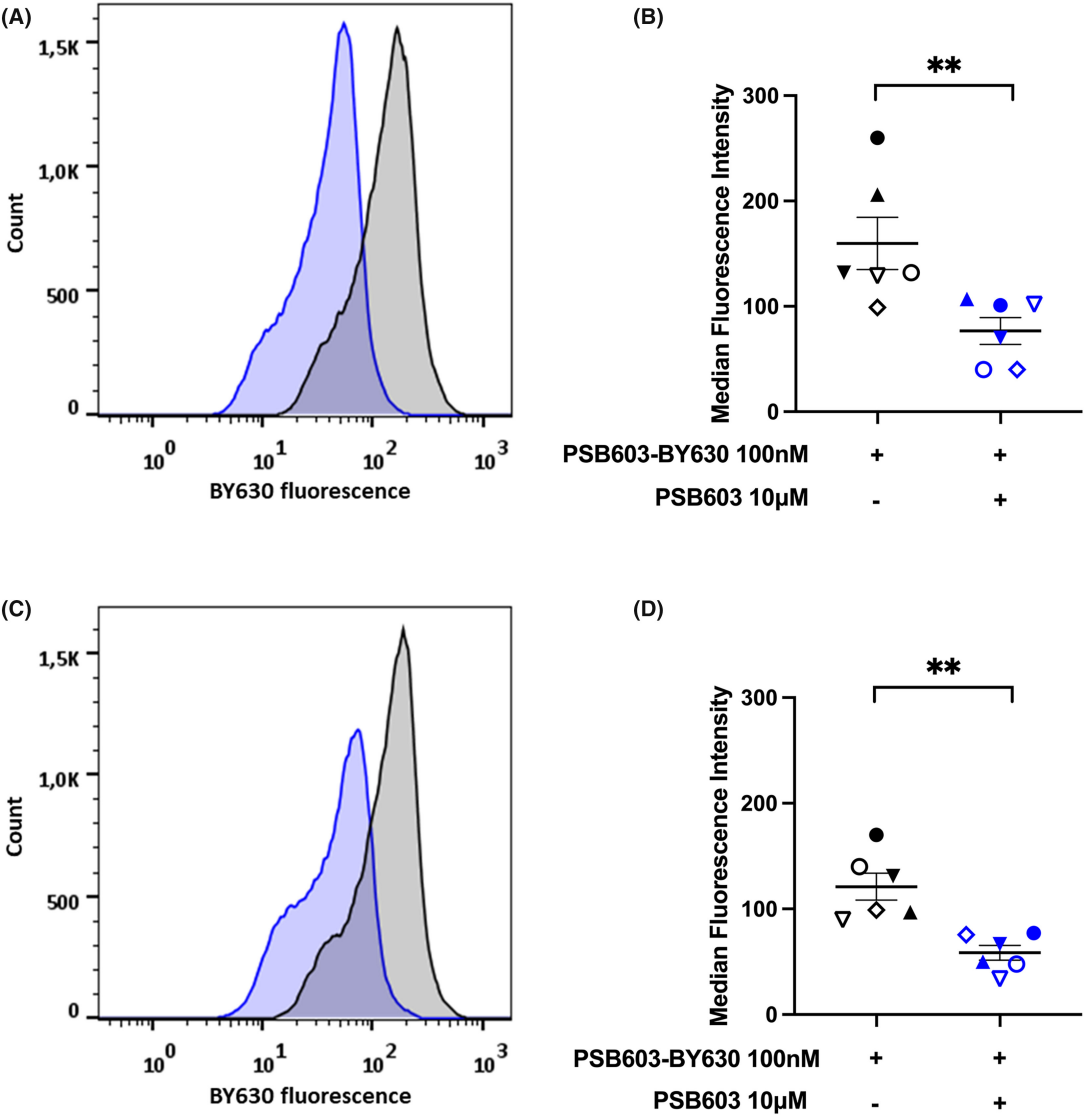
Flow cytometry data for the binding of 100 nM PSB603‐BY630 to endogenous A_2B_R in human monocyte‐derived M1‐like or M2‐like macrophages. (A) Flow cytometry histograms obtained in the absence and presence of the A_2B_‐selective antagonist PSB603 in a representative experiment from M1‐like macrophages differentiated from monocytes from a single representative donor. (B) Mean data for M1‐like macrophages obtained from six different donors in the presence or absence of 10 μM PSB603. Each symbol represents one donor and the lines show mean ± S.E.M. of the median fluorescence intensities (MFI). (C) Flow cytometry histograms obtained in the absence and presence of PSB603 in a representative experiment from M2‐like macrophages prepared from monocytes from a single donor (matched with the M1‐like data a‐b). (D) Mean data for M2‐like macrophages obtained from six different donors in the presence or absence of 10 μM PSB603. Each symbol represents one donor and the lines show mean ± S.E.M. of the MFIs. **p <.01 paired t‐test. (E) Specific binding (MFI, mean fluorescence intensity) of PSB603‐BY630 to M1‐like and M2‐like macrophages derived from the same donor. Specific binding was taken as the difference in MFI between total binding and that obtained in the presence of 10 μM PSB603 for each donor (taken from (B) and (D).

To evaluate the potential of this fluorescent ligand to monitor endogenous A_2B_R expression on human macrophages, we used flow cytometry to monitor specific PSB603‐BY630 binding in M1‐like and M2‐like macrophages. M1‐like and M2‐like macrophages were prepared from CD14+ human monocytes by differentiation (7 days) in macrophage medium containing GMCSF (20 U/mL) or MCSF (10 ng/mL) respectively. Macrophages were then labeled for 20 min (at room temperature) with 100 nM PSB603‐BY630 (in the presence or absence of 10 μM unlabelled PSB603) before being subjected to flow cytometry. Analysis of forward and side light scattering was used to gate out debris and exclude macrophage doublets (Figures S2,S3). Populations of singlet macrophages were then analyzed to generate histograms of cell count versus PSB603‐BY630 fluorescence intensity for M1‐like (Figure 8A) and M2‐like (Figure 8C) macrophages. Median fluorescence intensities obtained in M1‐like and M2‐like macrophages prepared from six independent donors are shown in Figure 8B,D respectively. Data from each donor were obtained in the presence and absence of unlabelled PSB603 (10 μM) and each symbol represents paired macrophages from a single donor. In both macrophage populations there was a significant inhibiton of PSB603‐BY630 binding by inclusion of 10 μM PSB603 to define non specific binding (*p* <.01; paired *t‐*test).

## 4 DISCUSSION

The present study reports on the properties of a new and selective red‐emitting fluorescent ligand (PSB603‐BY630) for the human A_2B_R. This molecule, together with the previously reported green‐emitting PSB‐12105,^31^ makes a good addition to existing fluorescent ligands which are selective for the A_2A_R^28^, ^29^ and opens the way to monitoring the endogenous expression levels of these two important adenosine receptors on immune cells. PSB603‐BY630 bound with high affinity (18.3 nM) to NLuc‐tagged A_2B_Rs stably expressed in HEK293G cells. The ligand exhibited very high selectivity for the A_2B_R with negligible specific‐binding detected to NLuc‐A_2A_R, NLuc‐A_1_R or NLuc‐A_3_R receptors at concentrations up to 500 nM. Competition binding studies demonstrated the expected pharmacology at A_2B_R with the A_2B_R‐selective ligands PSB603^4^, ^32^ and MRS‐1706^44^ demonstrating potent inhibition of the specific binding of 50 nM PSB603‐BY630. In contrast, selective A_2A_R‐antagonists SCH442416 and SCH58261,^45^, ^46^ The A_1_R‐selective antagonist SLV320^34^ and the A_3_R selective antagonist MRS1220^33^ were much lower affinity. Finally, kinetic studies undertaken at 37°C showed that equilibrium was reached within 60 min with 200 nM PSB603‐BY630 and analysis of the dissociation of the fluorescent ligand, initiated by addition of 10 μM MRS‐1706, allowed the residence time of PSB603‐BY630 to be determined as 15.4 min.

To establish whether the fluorescent variant of PSB603 still behaved as an A_2B_R antagonist in functional studies, we took advantage of the highly sensitive GloSensor biosensor for cAMP which is expressed in HEK293G cells and allows monitoring of functional responses mediated by A_2B_R and A_2A_R which are both endogenously expressed in these cells.^4^ Using the highly selective A_2B_‐selective agonist BAY 60–6583, we showed that PSB603‐BY630 was able to inhibit functional response to the A_2B_R‐selective agonist in HEK293G cells endogenously expressing A_2B_R. However, a striking feature of the antagonism produced by PSB603‐BY630 was that the main effect was a reduction of the maximal response to BAY 60–6583 with no significant effect on the agonist EC_50_ value. These data suggest a non‐competitive action of PSB603‐BY630. Furthermore, in a stable HEK293G cell line overexpressing recombinant human NLuc‐A_2B_Rs, the EC_50_ values for BAY 60–6583 were shifted to lower agonist concentrations consistent with an increase in the spare receptor reserve caused by A_2B_R overexpression. In these cells PSB603‐BY630 did produce a small increase in the EC_50_ for BAY 60–6583 but this was accompanied by a significant decrease in the maximal response to BAY 60–6583, again consistent with a non‐competitive interaction with BAY 60–6583 at the A_2B_R.

We have previously observed a similar non‐competitive effect of the parent compound PSB603 on BAY 60‐6583‐mediated GloSensor responses in HEK293G cells which was consistent with a negative allosteric effect of PSB603 at the A_2B_R.^4^ These data are consistent with a recent A_2B_R‐BAY60‐6583‐G_s_ cryo‐EM structure that revealed an orthosteric binding pocket for BAY60‐6583 that was similar to that of NECA, but with a secondary binding pocket extending out from the orthosteric binding site where residues V250^6.51^ and N273^7.36^ appear to be key determinants of its selectivity for A_2B_R.^14^ These data suggest that PSB603‐BY630 may also act as a negative allosteric regulator of the A_2B_R when coupled to Gs‐mediated responses.

A major driver for the generation of a selective red‐emitting fluorescent ligand for the A_2B_R was the need for a tool compound that could be used to monitor surface A_2B_R expression in individual immune cells. Recent studies have suggested that A_2B_Rs may regulate the immune response to the tumor microenvironment,^20^, ^21^, ^22^ in addition to the well‐established role of A_2A_Rs on immune cells in relation to cancer progression.^15^, ^16^, ^17^, ^18^, ^19^ As a first step towards this, we have used flow cytometry to monitor specific PSB603‐BY630 binding to A_2B_Rs on M1‐like and M2‐like human macrophages prepared from CD14+ monocytes from six different healthy donors. The data obtained show that this ligand can be used to detect endogenous A_2B_R expression in M1‐ and M2‐like macrophages. Given that individual cells will contain both specific (A_2B_R) and non‐specific binding sites, we chose to use median fluorescence intensity (MFI) to monitor the extent of A_2B_R receptor‐specific binding. Using this approach there was a significant (p < 0.01; paired t‐test) reduction in the total binding MFI measured with 100 nM PSB603‐BY630 in each donor in macrophages pre‐treated with 10 μM PSB603.

In summary, the present manuscript reports on the pharmacological properties of a new red‐emitting fluorescent ligand for the A_2B_R that has high affinity and selectivity. Furthermore, studies on M1‐ and M2‐like macrophages derived from CD14+ human monocytes have confirmed that PSB603‐BY630 can be used to monitor the endogenous expression of A_2B_R on immune cells. This ligand is an important addition to the library of fluorescent ligands, which are selective for each of the adenosine receptor subtypes, and should enhance the study of the role of adenosine receptors on immune cells in the tumor microenvironment.

## AUTHOR CONTRIBUTIONS

Participated in research design: HF, LEK, BK, SJH. Conducted experiments: FP, SJM, NDK, EC, JG. Performed data analysis: FP, SJH. Wrote or contributed to the writing of the manuscript: all authors.

## CONFLICT OF INTEREST STATEMENT

The authors declare no conflicts of interest.

## ETHICS STATEMENT

Heparinised whole blood was obtained by venepuncture from the antecubital fossa of the arm of healthy volunteers after written informed consent (Ethics from University of Nottingham Ethics committee, ref 161–1711).

## Supporting information


Data S1.

## Data Availability

The data that support the findings of this study are available from the corresponding author upon reasonable request.
