# Supplementary material for: A novel and selective fluorescent ligand for the study of adenosine A2B receptors
Source: Pharmacol Res Perspect. 2024 Jun 21;12(4):e1223. doi: 10.1002/prp2.1223 (PMC11191602; doi:10.1002/prp2.1223)
Supplement: Supplementary file 1 — Data S1. [file PRP2-12-e1223-s001.pdf]

Supplementary Information

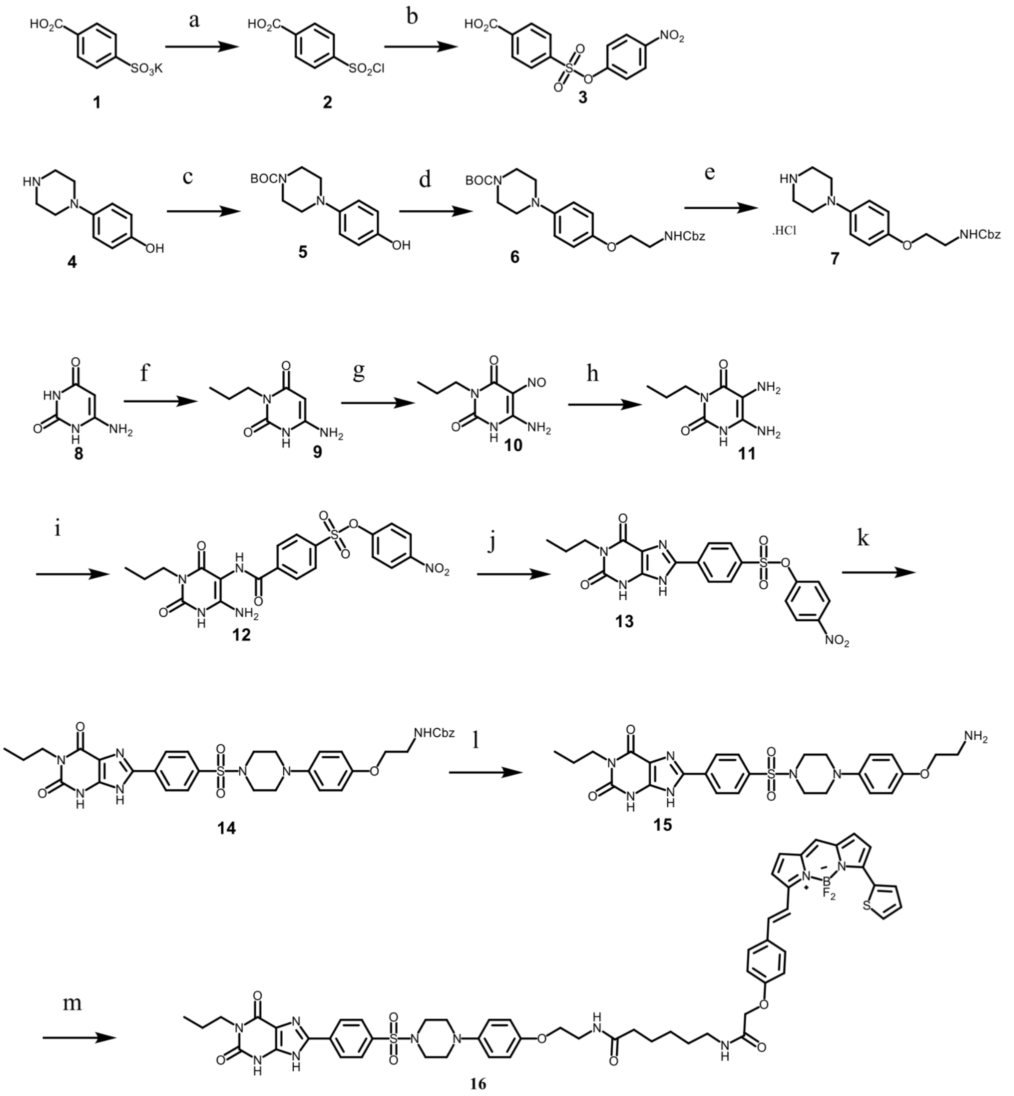

Supplementary Figure 1. Synthesis of PSB603-BY630. (a) Chlorosulfonic acid, room temperature overnight, then ice. (b) p-Nitrophenol THF, pH 8-9. (c) Boc_2-O, H_2O room temperature, 20 h. (d) $HOCH_2CH_2NHCbz$, DIAD, PPh_3. THF, 0°C to room temperature, 26 h. (e) 4 M HCl in dioxane, dioxane room temperature 3.5 h. (f) (i) HMDS, cat $(NH_4)_2SO_4$, reflux. (ii) 1-Iodopropane room temperature to 120°C 4 h. (g) $NaNO_2$, acetic acid, 70°C. (h) $Na_2S_2O_4$, NH_4OH, 70°C, 15 min. (i) Compound 3, EDC.HCl, MeOH, 20 h. (j) PPSE, 120°C for 10 min, 170°C for 3 h. (k) Compound 7, DIPEA, DMF, room temperature. (l) 10% Pd/C, H_2, DMF, room temperature, 2 h. (m) BODIPY-X-630/650-SE, DIPEA, DMF. **Abbreviations**; Boc_2O, di-*tert*-butyl dicarbonate; BODIPY 630/650-X-SE 6-(((4,4-difluoro-5-(2-thienyl)-4-

bora-*3a*,*4a*-diaza-*s*-indacene-3-yl)-stylryloxy)acetyl)aminohexanoic acid succinimidyl ester; DIPEA, *N,N*-diisopropylethylamine; DMF, *N,N*-dimethylformamide; DMSO, dimethylsulfoxide; EDC.HCl; *N*-(3-dimethylaminopropyl-*N*-ethylcarbodiimide hydrochloride, DIAD, diisopropylazodicarboxylate; HMDS, hexamethyldisilazane; PPSE, trimethylsilyl polyphosphate; THF, tetrahydrofuran.

Synthesis of PSB603-BY630. The synthesis of compound **16** was carried out in a convergent manner through the combination of three separate components. Firstly, to afford the sulfonate component (**3**), commercially available *p*-sulfobenzoic acid potassium salt (**1**) was treated with chlorosulfonic acid to give the sulfonyl chloride (**2**). Compound **2** was then treated with 4-nitrophenol to afford the sulfonate (**3**). Next 4-piperazin-1-yl phenol underwent *N*-Boc protection with Boc_2O to afford compound **5**. The phenol was then alkylated with *N*-*Z*-ethylenediamine to afford compound **6**. Compound **6** then underwent *N*-Boc deprotection in the presence of 2M HCl in 1,4-dioxane to afford the amine as hydrochloride salt form (**7**). Finally, to synthesise the xanthine component (**11**), 6-aminouracil (**8**) was first silylated with hexamethyldisilazane and then selectively *N*-alkylated at the 3-position using iodopropane. The resulting 3-propyl-6-aminouracil (**9**) was nitrosated utilising sodium nitrite and acetic acid to afford compound **10**, which was subsequently reduced using sodium dithionite to afford the diamine (**11**). Subsequently, compound **11** was then acylated with the sulfonate component (**3**) using EDC.HCl in methanol to afford the amide **12**. Compound 12 was then cyclised using trimethylsilyl polyphosphate (PPSE) with heating to afford compound **13**. To form the sulfonamide (**14**), compound **13** underwent treatment with the phenyl piperazine (**7**) and heating at reflux. Compound **14** was deprotected via catalytic hydrogenation using 10% palladium on carbon, to afford compound **15**. Finally, the congener (**15**) was labelled with 6-(((4,4-difluoro-5-(2-thienyl)-4-bora-*3a,4a*-diaza-*s*-indacene-3-yl)styryloxy)acetyl)amino hexanoic acid succinimidyl ester (BODIPY-630/650-X-SE) to afford the crude fluorescent ligand (**16**) which was purified by semi preparative HPLC. After lyophilisation, the identity of **16** was confirmed by HRMS (ES-TOF) and its purity (99%) by analytical HPLC.

4-(Chlorosulfonyl)benzoic acid (2)

To *p*-sulfobenzoic acid potassium salt (2.00 g, 8.32 mmol) at 0°C, chlorosulfonic acid (20 mL) was slowly added. The mixture was stirred overnight and then slowly poured onto ice-water. The precipitate was collected by vacuum filtration and dried under high vacuum to afford the title product as a white solid (1.60 g, 87%) which was used directly in the next step.

4-((*p*-Nitrophenoxy)sulfonyl)benzoic acid (**3**)

To compound 2 (1.00 g, 4.53 mmol), in a mixture of THF (25 mL) and Tris-HCl buffer (50 mM, 25 mL) *p*-nitrophenol (0.631 g, 4.53 mmol) in THF (25 mL) was added dropwise. The pH of

the reaction was kept at pH 8-9 by the addition 2M $NaOH_{(aq)}$. After stirring for 3 hours a further 0.1 eq of *p*-nitrophenol was added to the reaction mixture. After stirring for an additional hour, the pH was adjusted to pH 7 with 1M $HCl_{(aq)}$. The THF was removed *in vacuo* and the aqueous solution was acidified to pH 1. The precipitate was collected by vacuum filtration, washed with water and then dried under high vacuum. (0.761 g, 52 %). ^{1}H NMR (400 MHz, DMSO-d_6) δ = 13.73 (s, 1H), 8.27 (d, *J* = 9.1 Hz, 2H), 8.18 (d, *J* = 8.5 Hz, 2H), 8.04 (d, *J* = 8.5 Hz, 2H), 7.36 (d, *J* = 9.1 Hz, 2H). ^{13}C NMR (101 MHz, DMSO-d_6)) δ = 165.8, 152.9, 146.2, 137.1, 136.7, 130.7, 128.7, 125.9, 123.5.

***tert*-Butyl 4-(4-hydroxyphenyl)piperazine-1-carboxylate (5)**

To 4-piperazin-1-yl phenol (2.00 g, 11.2 mmol) in water (11.0 mL), Boc_2O (2.69 g, 12.3 mmol) was added. The mixture was stirred at room temperature for 19.5 hours and the solid that formed was collected by vacuum filtration. It was then washed with water and dried under high vacuum (2.88 g, 92%). ^{1}H NMR (400 MHz, DMSO) δ 8.86 (s, 1H), 6.79 (d, *J* = 8.9 Hz, 2H), 6.65 (d, *J* = 8.9 Hz, 2H), 3.43 (t, *J* = 5.0 Hz, 4H), 2.88 (t, *J* = 5.1 Hz, 4H), 2.50 (t, *J* = 1.9 Hz, 2H), 1.47 (s, 3H), 1.41 (s, 9H). ^{13}C NMR (101 MHz, DMSO) δ 153.8, 151.4, 144.0, 118.5, 115.4, 115.4, 78.9, 50.3, 40.2, 28.1, 26.9.

***tert*-Butyl 4-(4-(2-(((benzyloxy)carbonyl)amino)ethoxy)phenyl)piperazine-1-carboxylate (6)**

To compound 5 (1.43 g, 5.12 mmol) in THF (20.0 mL) at 0°C under N_2, PPh_3 (1.34 g, 5.12 mmol) and benzyl (2-hydroxyethyl)carbamate (1.00 g, 5.12 mmol) were added. DIAD (1.04 g, 5.12 mmol) was then added dropwise. The mixture was warmed to room temperature and was stirred for 26 hours. The solvent was removed and the crude product was loaded onto isolute before purification by column chromatography (4:6 EtOAc/pet. ether). The solvent was removed to afford a (1.43 g, 61%). ^{1}H NMR (400 MHz, DMSO) δ 7.43 – 7.23 (m, 1H), 6.98 – 6.71 (m, 1H), 5.03 (s, 0H), 3.91 (t, *J* = 5.8 Hz, 0H), 3.44 (t, *J* = 5.0 Hz, 1H), 2.94 (t, *J* = 5.1 Hz, 1H), 1.41 (s, 2H). ^{13}C NMR (101 MHz, DMSO) δ 156.2, 153.8, 152.4, 145.4, 137.1, 128.3, 127.8, 127.7, 118.0, 115.1, 78.9, 66.6, 65.3, 54.9, 49.8, 40.2, 28.1. LC-MS *m/z* calc calcd for $C_{25}H_{34}N_3O_5^+$ [M+H]$^+$; 456 found; 456.0, t_R = 3.01 min.

Benzyl (2-(4-(piperazin-1-yl)phenoxy)ethyl)carbamate (7)

To compound 6 (0.200 g, 0.44 mmol) in dioxane (2.0 mL), 4M HCl in dioxane (2.0 mL) was added. The mixture was stirred at room temperature for 3.5 hours and the solvent was removed under high vacuum. The resulting white foam was used directly in the next step. LC-MS *m/z* calc calcd for $C_{20}H_{26}N_3O_3^+$ [M+H]$^+$; 356 found; 355.7, t_R = 2.12 min.

3-Propyl-6-aminouracil (9)

A mixture of 6-aminouracil (12.7 g, 0.1 mol), hexamethyldisilazane (HMDS) (21.02g, 0.13 mol , 27.3 ml) and catalytic ammonium sulphate (0.1g) was heated at reflux for 24hr. After cooling, more HMDS (30ml) was added and the mixture heated for another 24hr. After cooling, the mixture was evaporated in vacuo to a thick slurry (about 30ml had evaporated). The slurry was stirred and iodopropane (33.7 g, 0.2 mol, 19.4 ml) added slowly at rt over 10 minutes, no exotherm. After 0.5hr the mixture was heated at 60°C for 0.5hr then 120°C for 3hrs. After cooling, water was added and the mixture stirred. A precipitate formed which was collected by filtration, washing with water, toluene and diethyl ether. The solid was suspended in methanol (50ml), filtered, dried and collected. Yield 7.0g, 0.041 mol.

^{1}H NMR (400 MHz, DMSO-d_6) δ = 10.31 (s, 1H), 6.16 (s, 2H), 4.53 (s, 1H), 3.60 (t, J = 7.6 Hz, 2H), 1.46 (sextet, J = 7.4 Hz 2H), 0.80 (t, J = 7.5 Hz, 3H). ^{13}C NMR (101 MHz, DMSO-d_6)) δ 163.0, 153.5, 151,0, 74.2, 21.0, 11.2. LC-MS *m/z* calc. for calcd for $C_7H_{12}N_3O_2^+$ [M+H]$^+$; 170.1, found; 170.4, t_R = 1.18 min.

3-Propyl-5-nitroso-6-aminouracil (10)

To compound 9 (0.30 g, 1.77 mmol) in 50% acetic acid (9.0 mL) at 70°C, sodium nitrite (0.36 g, 3.55 mmol) was added portion-wise. The clear colourless solution turned purple and a yellow-orange precipitate then formed. The precipitate was collected by vacuum filtration, washed with water and then dried under high vacuum (0.31 g, 87%). ^{1}H NMR (400 MHz, DMSO-d_6) δ 11.44 (s, 2H), 7.96 (s, 1H), 3.80 (t, J = 7.2 Hz, 2H), 1.60 (sextet, J = 7.4 Hz, 2H), 0.89 (t, J = 7.5 Hz, 3H). ^{13}C NMR (101 MHz, DMSO-d_6)) δ = 161.1, 149.0, 144.3, 139.8, 41.3, 20.8, 11.2. LC-MS *m/z* calc. for calcd for $C_7H_{11}N_4O_3^+$ [M+H]$^+$; 199.1 found; 199.3, t_R = 0.80 min.

5,6-Diamino-3-propyluracil (11)

Compound 10 (0.700g, 3.53 mmol) was added to $NH_4OH_{(aq)}$ and then heated to 70°C. $Na_2S_2O_4$ (1.845 g, 10.6 mmol) was added portion-wise and the pink solution decolourised. The mixture was stirred for 15 minutes and then the solution was concentrated until crystals appeared. The mixture was cooled in the fridge and then the crystals were collected by vacuum filtration and washed with cold water. The product (0.595 g, 91%) was used directly in the next step. ^{1}H NMR (400 MHz, DMSO-d_6) δ 9.50-10.50 (br s, 1H), 5.55 (s, 2H), 3.70 – 3.62 (m, 2H), 2.60-3.20 (br s, 2H), 1.49 (h, J = 7.4 Hz, 2H), 0.82 (t, J = 7.4 Hz, 3H).

4-nitropheny-4-((6-amino-2,4-dioxo-3-propyl-1,2,3,4-tetrahydropyrimidin-5-yl)carbamoyl)benzenesulfonate (12)

To compound 11 (68.0 mg, 0.37 mmol) in MeOH (4.0 mL) compound 3 (0.11 g, 0.37 mmol) and EDC (78.0 mg, 20.41 mmol) were added. The mixture was stirred at room temperature for 20 hrs and water was added. A yellow precipitate formed which was collected by vacuum filtration and the washed with water. The solid was dissolved in a minimal volume of DMF and then precipitated out with water. The solid was collected by vacuum filtration and the dried under high vacuum (0.149 g, 78%). ^{1}H NMR (400 MHz, DMSO-d_6) δ 10.53 (s, 1H), 9.23 (s, 1H), 8.29 (d, *J* = 9.1 Hz, 2H), 8.19 (d, *J* = 8.3 Hz, 2H), 8.04 (d, *J* = 8.5 Hz, 2H), 7.39 (d, *J* = 9.1 Hz, 2H), 6.23 (s, 2H), 3.66 (t, *J* = 7.1 Hz, 2H), 1.50 (sextet, *J* = 7.4 Hz, 2H), 0.83 (t, *J* = 7.4 Hz, 3H). LC-MS *m/z* calc calcd for $C_{20}H_{18}N_5O_8S^-$ [M-H]$^-$; 488 found; 488.2, t_R = 2.45 min.

1-Propyl-8-[4-[[[p-nitrophenyl]oxy]sulfonyl]phenyl]xanthine (13)

To PPSE (0.5 mL), compound 12 (0.14 g, 0.30 mmol) was added. The mixture was heated at 120°C for 10 mins and then 170°C for 3 hrs. The reaction mixture was cooled to room temperature and the white solid was collected by vacuum filtration and washed with methanol. (92.0 mg, 67%). ^{1}H NMR (400 MHz, DMSO-d_6) δ 14.15 (s, 1H), 12.00 (s, 1H), 8.35 (d, *J* = 8.4 Hz, 2H), 8.28 (d, *J* = 9.1 Hz, 2H), 8.05 (d, *J* = 8.4 Hz, 2H), 7.39 (d, *J* = 9.1 Hz, 2H), 3.82 (t, *J* = 7.4 Hz, 2H), 1.58 (h, *J* = 7.2 Hz, 2H), 0.88 (t, *J* = 7.4 Hz, 3H). ^{13}C NMR (101 MHz, DMSO-d_6) δ 154.9, 153.0, 150.9, 147.6, 147.3, 146.2, 134.9, 134.1, 129.1, 127.3, 125.9, 123.5, 108.8, 41.5, 20.9, 11.2. LC-MS *m/z* calc calcd for $C_{20}H_{18}N_5O_7S^+$ [M+H]$^+$; 472.1 found; 472.3, t_R = 2.77 min.

Benzyl (2-(4-(4-((4-(2,6-dioxo-1-propyl-2,3,6,9-tetrahydro-1H-purin-8-yl)phenyl)sulfonyl)piperazin-1-yl)phenoxy)ethyl)carbamate (14)

To compound 7 (0.19 g, 0.44 mmol) in DMF (2.0 mL), DIPEA (0.15 mL, 0.88 mmol) was added compound 13 (42mg, 0.088mmol). The mixture was stirred at room temperature and as no reaction had occurred it was then heated at reflux for 1 hour. The solvent was removed under vacuum to afford a thick brown residue which was purified by column chromatography (3:97 MeOH/DCM) after loading onto isolute. The product was then purified further by semi-preparative HPLC (40 to 95% MeCN over 14 mins, with a 2 min hold at 60% MeCN at 10 mins). To remove any residual formic acid from the product after lyophilisation, the solid was dissolved in DMF and Amberlyst A21 resin was added. The mixture was stirred slowly for 10 mins then filtered. The solvent was removed to afford the title product as an off-white solid (14.7 mg, 24%). ^{1}H NMR (400 MHz, DMSO) δ 8.23 (d, *J* = 8.4 Hz, 2H), 7.69 (d, *J* = 8.3 Hz, 2H), 7.43 (t, *J* = 5.8 Hz, 1H), 7.42 – 7.21 (m, 6H), 6.88 – 6.73 (m, 4H), 3.88 (t, *J* = 5.8 Hz, 2H), 3.78 (t, *J* = 7.5 Hz, 2H), 3.31 (q, *J* = 5.6 Hz, 2H), 3.22 – 2.85 (m, 8H), 1.53 (q, *J* = 7.4 Hz, 2H), 0.86 (t, *J* = 7.4 Hz, 3H). LC-MS *m/z* calc calcd for $C_{35}H_{37}N_7O_7S^+$ [M+H]$^+$; 688.2 found; 688.0, t_R = 2.86 min.

8-(4-((4-(4-(2-aminoethoxy)phenyl)piperazin-1-yl)sulfonyl)phenyl)-1-propyl-3,9-dihydro-1H-purine-2,6-dione (15)

To compopund 14 (9.8 mg, 14.2 µmol) in DMF (2.0 mL), 10% Pd on carbon (1.0 mg) was added. The flask was evacuated and then filled with H_2. This was repeated twice. After stirring for 2 hours the mixture was filtered through celite and the solvent was removed from the filtrate to afford the title product as an off white solid (4.9 mg, 61%) which was used directly in the next step. LC-MS *m/z* calc calcd for $C_{26}H_{31}N_7O_5S^+$ $[M+H]^+$; 554.2 found; 554.0, t_R = 2.13 min.

(E)-6-(2-(4-(2-(5,5-difluoro-7-(thiophen-2-yl)-5H-4□^{4},5□^{4}-dipyrrolo[1,2-c:2',1'-f][1,3,2]diazaborinin-3-yl)vinyl)phenoxy)acetamido)-N-(2-(4-(4-((4-(2,6-dioxo-1-propyl-2,3,6,9-tetrahydro-1H-purin-8-yl)phenyl)sulfonyl)piperazin-1-yl)phenoxy)ethyl)hexanamide (16)

To compound 15 (1.3 mg, 2.27 µmol) in DMF (0.2 mL), DIPEA (1.3 µL, 7.57 µmol) was added. The mixture was stirred for 5 minutes then BODIPY-X-630/650-SE (1.0 mg, 1.51 µmol) in DMF (0.8 mL) was added. The mixture was stirred at room temperature in the absence of light for 4.25 hours and the solvent was then removed. The crude product was purified by semi-preparative HPLC (60% to 65% MeCN over 10 minutes) to afford a blue solid after lyophilisation (1.4 mg, 82%). HRMS (ESI-TOF) *m/z* calcd for $C_{55}H_{58}BF_2N_{10}O_8S^+$ $[M+H]^+$; 1098.3863 found; 1121.3708 $[M+Na]^+$. Analytical RP-HPLC; t_R = 19.22 mins, purity = 99%

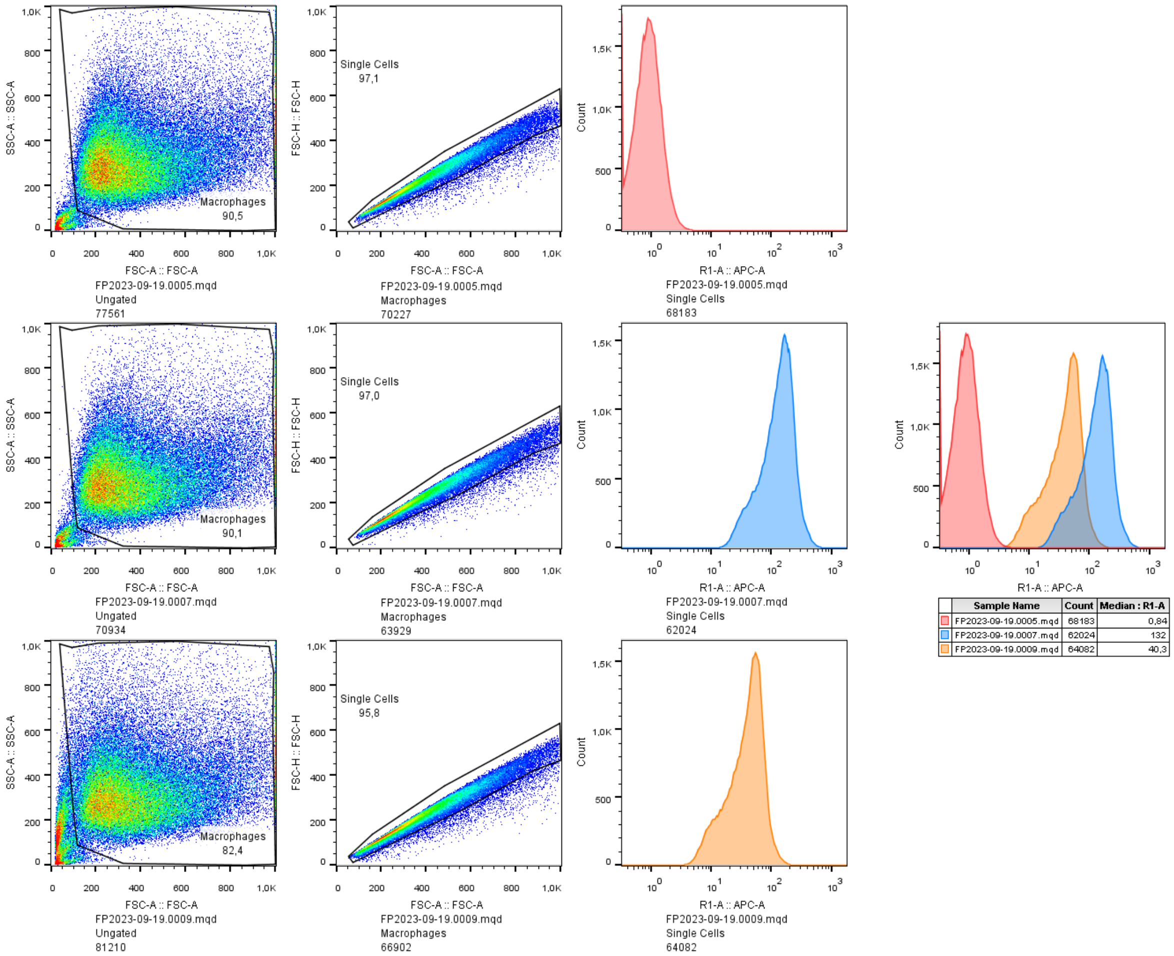

Supplementary Figure 2. Gating strategy of flow cytometry of human monocyte derived M1-like macrophages. Left panels show dot plots of acquired cells based on their size (Forward Scatter Area; FSC-A) and their granularity (Side Scatter Area; SSC-A) where a gate is set to include macrophages and exclude dead cells and debris. Gated macrophages are then plotted in the middle dot plots (Forward Scatter Area vs Forward Scatter-Height) in order to exclude doublets. Single cell macrophages are then presented as histograms in the right panels where fluorescence intensity is plotted against cell count. The three top graphs correspond to the unstained sample, middle row graphs correspond to the sample stained with PSB603-BY630 only and the three bottom graphs display the sample preincubated with PSB603 and then stained with PSB603-BY630. The single graph on the far right represents a merge of the three samples displaying overlaid histograms.

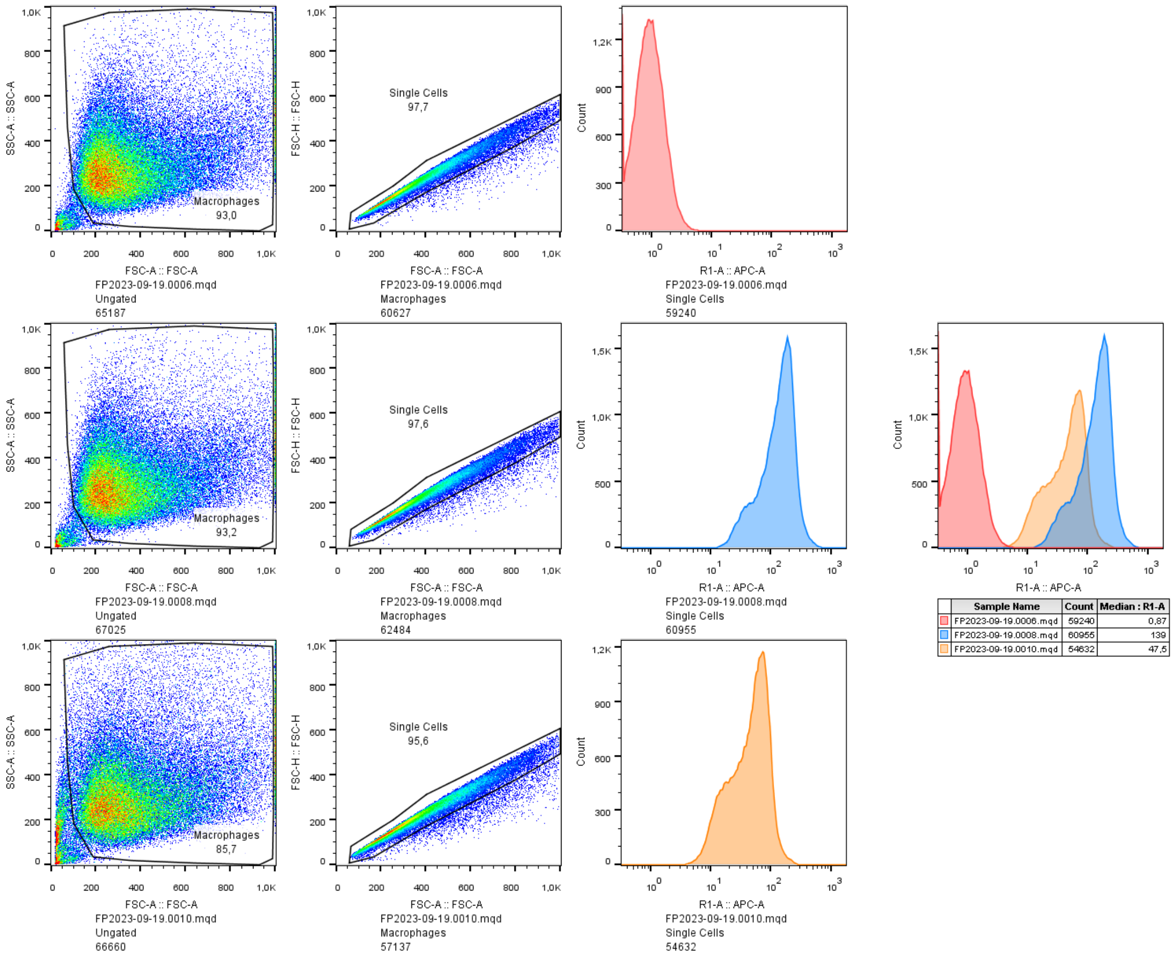

Supplementary Figure 3. Gating strategy of flow cytometry of human monocyte derived M2-like macrophages. Left panels show dot plots of acquired cells based on their size (Forward Scatter Area; FSC-A) and their granularity (Side Scatter Area; SSC-A) where a gate is set to include macrophages and exclude dead cells and debris. Gated macrophages are then plotted in the middle dot plots (Forward Scatter Area vs Forward Scatter-Height) in order to exclude doublets. Single cell macrophages are then presented as histograms in the right panels where fluorescence intensity is plotted against cell count. The three top graphs correspond to the unstained sample, middle row graphs correspond to the sample stained with PSB603-BY630 only and the three bottom graphs display the sample preincubated with PSB603 and then stained with PSB603-BY630. The single graph on the far right represents a merge of the three samples displaying overlaid histograms.
